# Advances in Machine Learning-Assisted Optical Sensing Arrays for Disease Diagnosis

**DOI:** 10.3390/biomimetics11080531

**Published:** 2026-07-31

**Authors:** Xuetong Sun, Hao Sun, Beibei Wang, Huaishu Lin

**Affiliations:** 1 College of Life Sciences, Zhuhai College of Science and Technology, Zhuhai 519040, China; 2 College of Chemistry, Jilin University, Changchun 130012, China; 3 Department of Economics and Management, Guangdong Technical College of Water Resources and Electric Engineering, Guangzhou 510925, China

**Keywords:** optical sensing arrays, machine learning, disease diagnosis, colorimetric detection, fluorescent sensor arrays, surface-enhanced Raman scattering

## Abstract

Optical sensing arrays have emerged as transformative tools for disease diagnosis, offering low-cost, rapid, and multiplexed fingerprint detection capabilities. However, the high-dimensional and complex data generated by these arrays pose significant challenges for conventional analytical methods. The integration of machine learning (ML) has advanced this field by enabling automated feature extraction, robust pattern recognition, and accurate disease classification. This review provides a systematic overview of ML-reinforced optical sensing arrays, with a particular focus on three major modalities: colorimetric, fluorescent, and surface-enhanced Raman scattering (SERS) sensor arrays. We critically evaluate how ML algorithms—encompassing unsupervised, supervised, and deep learning paradigms—synergistically enhance the diagnostic performance of each sensor modality. Representative applications are highlighted, demonstrating high accuracy in detecting cancers and infectious diseases. Finally, we discuss the pressing challenges related to data standardization, model interpretability, and clinical translation, while outlining future directions toward intelligent, point-of-care, and personalized diagnostic systems.

## 1. Introduction

Diseases, particularly life-threatening conditions such as malignancies, cardiovascular disorders, and acute infectious diseases, impose enormous burdens on global healthcare systems. According to the Global Burden of Disease Study, non-communicable diseases alone account for over 70% of annual deaths worldwide, while emerging and re-emerging pathogens continue to challenge public health infrastructures [1]. For many of these diseases, early and accurate diagnosis is the cornerstone of effective intervention—it directly determines therapeutic windows, influences prognosis, and reduces healthcare costs. In oncology, for instance, five-year survival rates for patients diagnosed at stage I versus stage IV differ by orders of magnitude for most solid tumors [2]. Similarly, rapid identification of bacterial or viral pathogens in sepsis or respiratory infections can reduce mortality and curb transmission [3].

Conventional laboratory-based diagnostic techniques, including blood and urine analysis combined with medical imaging, remain the cornerstone of disease diagnosis [4,5,6]. However, these methods suffer from several limitations: complex and expensive instrumentation, high demands on operator expertise, substantial equipment maintenance costs, and prolonged turnaround times that may delay effective treatment. Consequently, there is an urgent need to develop low-cost, rapid, convenient, and highly accurate diagnostic technologies.

Optical sensing arrays have emerged as a transformative paradigm to address these challenges [4,7,8]. By leveraging cross-reactive optical sensing elements, such arrays generate multidimensional response patterns—akin to the biological coding mechanism of the mammalian olfactory system. This biomimetic design facilitates multiplexed, fingerprint-like discrimination of complex biological samples. The resulting high-dimensional datasets are inherently well-suited for ML-based analysis, enabling robust extraction of subtle, discriminative features that support accurate disease classification [9,10]. In contrast to conventional “lock-and-key” biosensors—which rely on highly specific, one-analyte–one-receptor binding—optical sensing arrays exploit differential, ensemble-level interactions across multiple sensing elements to produce rich, multivariate optical signatures. This capability allows for the simultaneous identification and quantification of multiple analytes within heterogeneous biological matrices.

**Figure 1:**
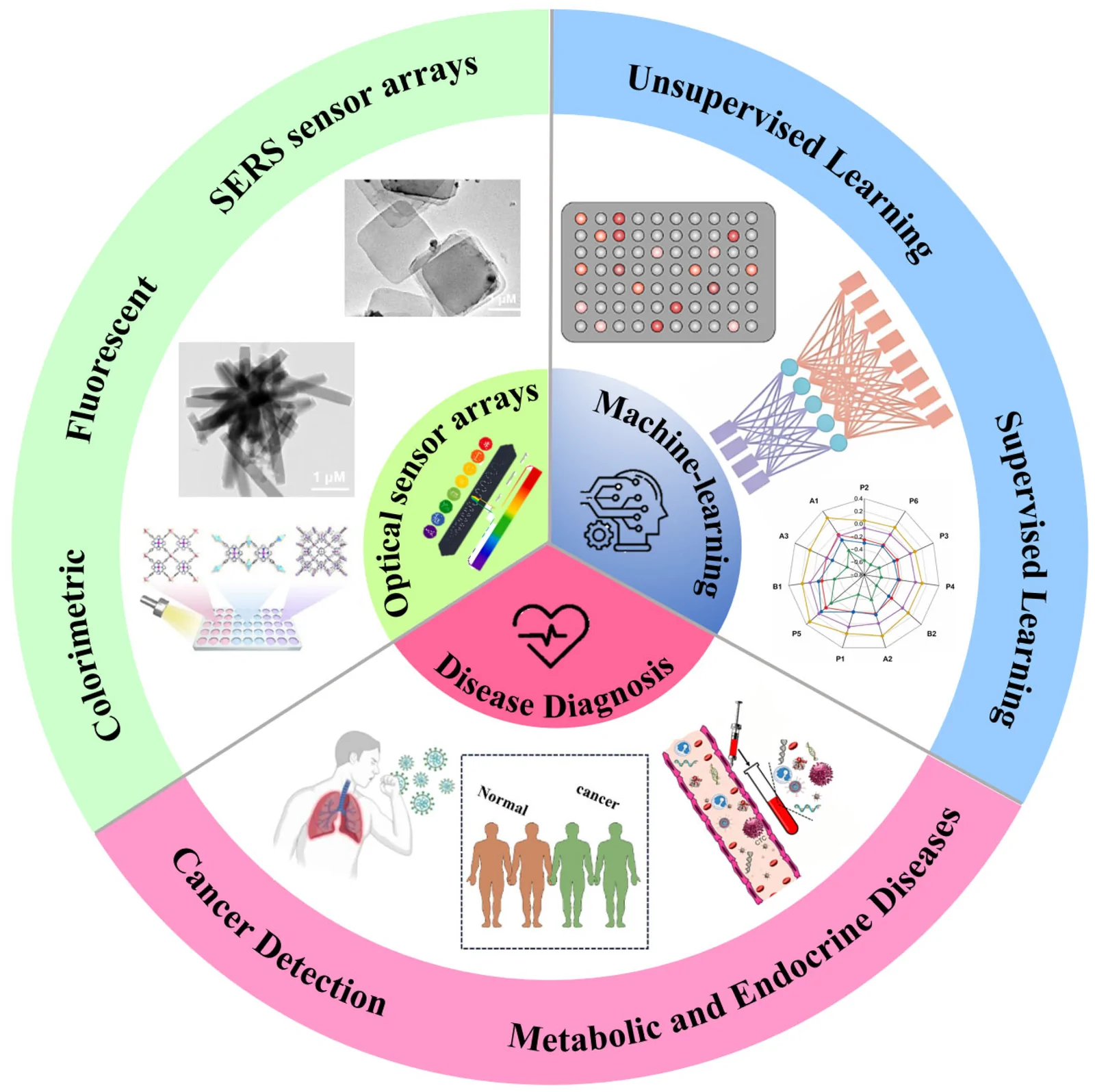
Schematic overview of ML-assisted optical sensing arrays for disease diagnosis.

As schematically illustrated in Figure 1, the major optical sensing array modalities include colorimetric, fluorescence-based arrays, and SERS sensors. These platforms generate characteristic optical fingerprints upon exposure to disease-related biomarkers, allowing the discrimination of normal versus pathological states. The figure highlights their application in two broad diagnostic areas: cancer detection (e.g., differentiating malignant from benign lesions) and metabolic/endocrine disease diagnosis. However, the practical application of these arrays faces a critical bottleneck: the large volume of multidimensional data they generate. A typical array may produce hundreds to thousands of data points per sample, encompassing spectral intensities, color channels, or temporal dynamics [9]. Traditional univariate or simple multivariate statistical methods often fail to extract the subtle, non-linear patterns that distinguish different disease states or biomarker profiles. This is where ML becomes indispensable. As a subset of artificial intelligence, ML algorithms are designed to automatically learn complex relationships from data without requiring explicit programming of rules [10]. Unsupervised techniques, such as principal component analysis (PCA), t-distributed stochastic neighbor embedding (t-SNE), and hierarchical cluster analysis (HCA), enable dimensionality reduction and pattern discovery, facilitating data visualization and hypothesis generation [11,12,13]. Supervised algorithms, including support vector machines (SVMs), random forests (RFs), artificial neural networks (ANNs), and deep learning architectures, can be trained on labeled datasets to accurately classify unknown samples, quantify analyte concentrations, and even predict disease progression [14,15]. By integrating ML into the sensing workflow, researchers can transform raw optical fingerprints into clinically actionable diagnostic decisions, enhancing accuracy, reproducibility, and throughput.

Recent years have witnessed a surge in reports combining ML with optical sensor arrays for disease diagnosis, covering cancers, infectious diseases, metabolic disorders, and neurodegenerative conditions [16,17,18]. For example, ML-assisted colorimetric nanozyme arrays have realized >95% accuracy in urinary tract infection diagnosis [19], while deep learning-enhanced SERS platforms have discriminated breast cancer subtypes from serum exosomes with high classification accuracy [20]. Fluorescent sensor arrays coupled with DNA computing or aggregation-induced emission probes have enabled rapid pathogen identification and antibiotic resistance profiling [21,22]. Although several review articles have summarized ML-assisted biosensors [23] and sensor arrays for various applications [13,24], a comprehensive review that systematically compares the synergistic integration of ML algorithms with different optical sensing modalities—specifically colorimetric, fluorescent, and SERS arrays—for disease diagnosis remains relatively limited.

This review aims to fill that gap by providing a systematic overview of ML-reinforced optical sensing arrays for disease diagnosis. Specific attention is given to colorimetric, fluorescent, and SERS sensor arrays, as these three modalities currently dominate the ML-assisted diagnostic literature and exhibit the greatest clinical translation maturity. Other optical sensing principles—including chemiluminescence, surface plasmon resonance (SPR), interferometric, and terahertz arrays—have shown considerable biosensing promise but remain underrepresented in ML-integrated array platforms for diagnostic applications. The fundamental principles of optical sensor arrays and commonly used ML algorithms are first introduced. Representative applications are then categorized and critically evaluated for the three major array types, with emphasis on how ML enhances each modality’s diagnostic performance. Disease-specific applications, such as cancer and infectious diseases, are further discussed. Current challenges related to data quality, model interpretability, and clinical translation are addressed, and future directions toward intelligent, portable, and personalized diagnostic systems are proposed. Through this comprehensive synthesis, it is hoped that a clear roadmap will be provided for researchers and clinicians to harness the power of ML and optical sensing arrays in next-generation disease diagnostics.

A literature search was conducted using the Web of Science, PubMed, and Scopus databases for articles published between January 2016 and July 2026. The search terms included combinations of “optical sensing array,” “machine learning,” “colorimetric,” “fluorescent,” “surface-enhanced Raman scattering,” and “disease diagnosis.” Only peer-reviewed original research and review articles written in English were considered.

## 2. Overview of Optical Sensing Arrays

Optical sensing arrays mimic human taste recognition mechanisms to identify target analytes [25]. Each sensor array consists of multiple sensing units, with each unit exhibiting selective or cross-reactive responses to specific analytes. When analytes adsorb or bind to sensor surfaces, the optical signals of these sensing units change accordingly. By analyzing these optical signal patterns and comparing them with known standards, information about analyte types and concentrations can be obtained, facilitating both qualitative and quantitative analysis [26,27].

However, cross-sensitivity or interference between different sensing units may occur—a single sensing unit might respond to multiple analytes, potentially causing detection errors. Additionally, optical sensing arrays generate multidimensional signal data that require sophisticated data analysis and pattern recognition algorithms. Therefore, constructing simple yet stable automated detection models is essential for achieving precise disease diagnosis.

Based on signal output types, optical sensing arrays can be classified into three main categories: Colorimetric sensor arrays detect color changes resulting from chemical reactions or physical adsorption between target analytes and sensing elements [19,28,29]. These sensors offer advantages including rapid response, intuitive results, and low cost. Color changes can be quantified using UV-Vis spectrophotometers or microplate readers, and ML algorithms subsequently analyze absorbance data for accurate disease diagnosis. Fluorescent sensor arrays employ multiple fluorescent sensing units to attain high sensitivity and specificity [29,30,31,32]. The detection principle relies on specific interactions between fluorescent probes and target molecules, causing changes in the microenvironment around fluorophores and consequently altering fluorescence intensity, lifetime, or polarization parameters. These fluorescence parameter variations correlate closely with analyte types and concentrations. SERS sensor arrays combine high sensitivity with molecular fingerprinting capabilities, making them particularly valuable for biomarker detection and disease diagnosis [33,34,35]. SERS sensors can be further divided into label-free and labeled types, with ML algorithms effectively processing complex SERS spectral data for improved diagnostic efficiency and accuracy.

Compared to traditional single-target detection methods, optical sensing arrays offer several distinct advantages: First, they enable multiplex detection, simultaneously analyzing multiple biomarkers from a single sample. Second, they tolerate imperfect selectivity—instead of requiring highly specific recognition elements, they rely on cross-reactive response patterns. Third, they provide rapid analysis, with detection completed within minutes to an hour. Fourth, they offer cost-effective implementation, often using inexpensive materials, and are compatible with smartphone-based readouts. Finally, they can be miniaturized for point-of-care applications.

## 3. ML Algorithms

In the current data-driven era, the proliferation of wearable monitors, high-throughput screening platforms, and real-time diagnostic tools has generated an unprecedented volume of complex, multi-dimensional health-related data. Optical sensing arrays, with their multiplexed and time-resolved output, are a prime example of such data-rich technologies. ML provides a powerful framework for navigating this data landscape—not by presetting rigid analytical rules but by learning intrinsic structures and correlations directly from the measured signals [36]. In biosensor development, ML has become an indispensable tool for processing and interpreting the vast information embedded in optical fingerprints [37,38].

**Figure 2:**
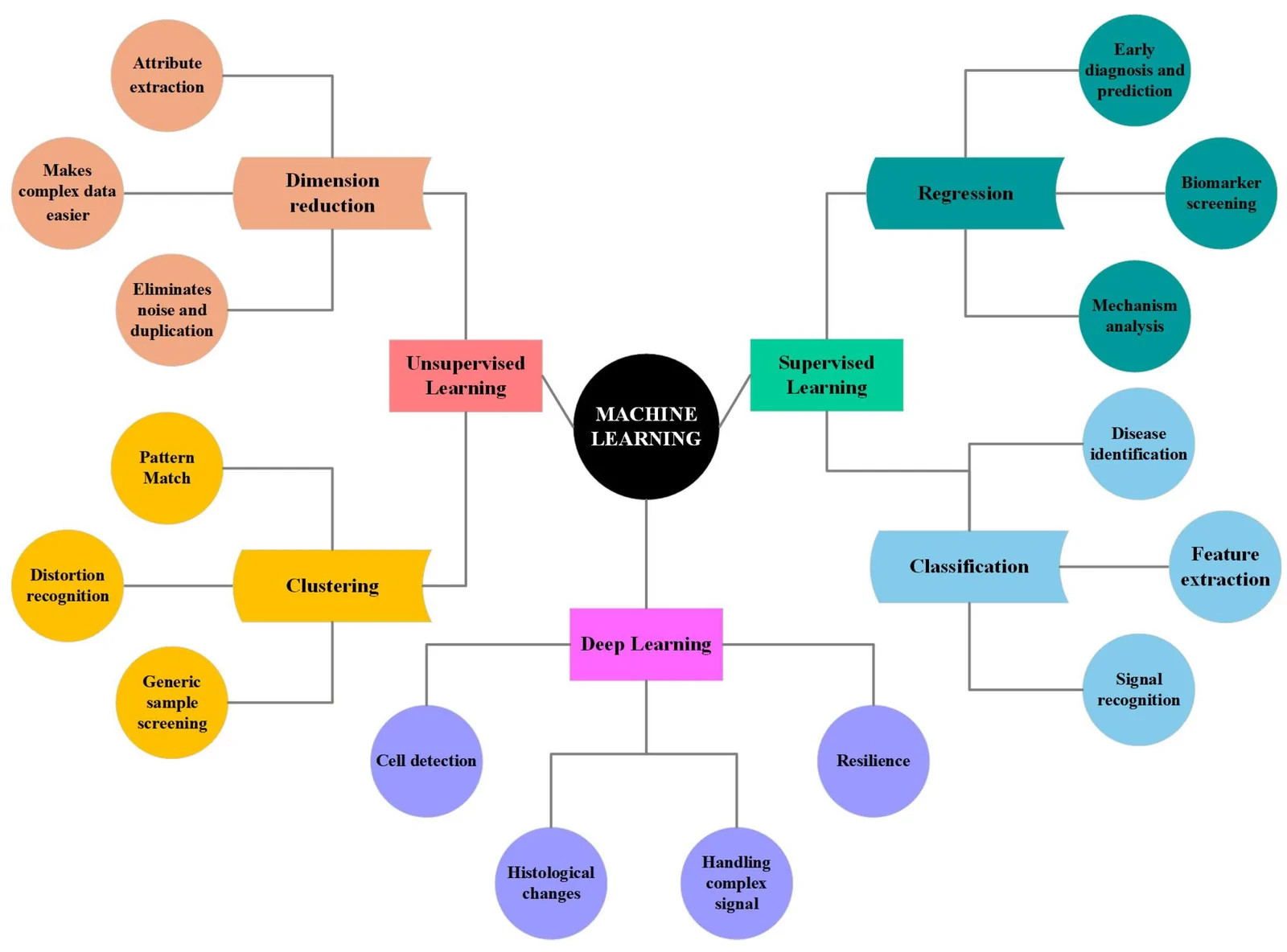
Schematic overview of ML approaches applied to optical sensing arrays.

Figure 2 illustrates a hierarchical overview of ML approaches commonly applied to optical sensing arrays. The taxonomy categorizes algorithms into three main families—unsupervised, supervised, and deep learning—each with specific sub-categories and representative techniques. Unsupervised methods (e.g., PCA, t-SNE, UMAP, HCA, and K-means) are primarily used for dimensionality reduction and clustering, enabling data visualization and discovery of hidden disease subtypes. Supervised algorithms (e.g., SVM, RF, ANN, k-NN, and regression models) are designed for classification and quantification, using labeled datasets to predict disease states or biomarker concentrations. Deep learning architectures (e.g., CNN, RNN/LSTM, and emerging models like KAN) automatically extract hierarchical features from raw spectral or imaging data, often reaching state-of-the-art performance in complex diagnostic tasks. This mapping highlights the central argument of the review: ML enhances optical biosensor performance by improving accuracy, sensitivity, and clinical decision-making in healthcare.

### 3.1. Unsupervised Learning Algorithms

Unsupervised learning operates on unlabeled datasets, aiming to discover hidden structures, patterns, or relationships without any predefined output labels [9,39,40]. In optical sensor array analysis, unsupervised methods are primarily employed for two purposes: dimensionality reduction and clustering.

Dimensionality reduction reduces the number of variables while retaining the most informative features, thereby mitigating the “curse of dimensionality” and facilitating visualization. PCA is a linear technique that projects high-dimensional data onto a lower-dimensional space by preserving the directions of maximum variance. PCA is widely used for preprocessing spectral or colorimetric data, as it quickly reveals natural groupings and outliers [41,42]. For instance, in SERS-based serum profiling, PCA has been used to separate cancer patients from healthy controls prior to supervised classification. However, PCA is sensitive to outliers and assumes linear correlations, which may not hold for complex biological systems.

t-SNE is a non-linear method that maps high-dimensional points to a low-dimensional space while preserving local neighborhood structures. For instance, Yaltaye et al. employed t-SNE to visualize single-molecule SERS spectra of serine and tyrosine phosphorylation, which allowed clear separation of phospho-isoforms after k-means-based data cleaning [43]. Similarly, Rodriguez-Nieves et al. used t-SNE to project deconvoluted multicolor SERS data onto a 2D plane, realizing distinct clustering of three breast cancer cell lines based on integrin expression profiles [44]. Nevertheless, t-SNE is stochastic and computationally intensive, and its results require careful interpretation due to lack of reproducibility across runs.

Uniform Manifold Approximation and Projection (UMAP) is a more recent non-linear technique that balances local and global structure preservation. UMAP has gained popularity in analyzing multi-omics and biosensor data, particularly when combined with clustering algorithms for downstream analysis. For instance, García-Astrain et al. employed UMAP to visualize over 13,000 time-series SERS spectra from a 3D cancer cell model, followed by k-means clustering to identify distinct temporal clusters reflecting extracellular matrix remodeling [45]. Similarly, Fugang Liu et al. utilized UMAP for dimensionality reduction in single-cell SERS metabolic profiles after random forest feature selection, enabling clear separation of HeLa and MCF-7 cancer cell lines, with hierarchical clustering further confirming distinct metabolic phenotypes [46].

Clustering groups data points based on similarity metrics, thereby facilitating the discovery of natural categories or disease subtypes. HCA builds a dendrogram by iteratively merging or splitting clusters according to distance measures. It is useful for exploratory analysis of sensor responses, such as differentiating bacterial strains or tumor exosome profiles, because it does not require a predefined number of clusters [13,47]. However, HCA is sensitive to noise and linkage choices, and it can be computationally expensive for large datasets.

K-means clustering partitions data into a fixed number (k) of clusters by minimizing within-cluster variance. It is simple, efficient, and well-suited for large-scale datasets, such as time-series signals from wearable sensors or multiplexed fluorescence patterns. For example, Lu et al. employed K-means clustering to classify the output signals of a FRET cascade miRNA addition probe, achieving 100% accurate diagnosis of acute myocardial infarction [48]. Joshi and Mondal applied K-means clustering to analyze single-molecule fluorescence data from an influenza A disease model, which permitted the estimation of critical biophysical parameters such as cluster density and area for viral protein assembly [49]. The main drawbacks are the need to predefine k and sensitivity to initial centroids, often mitigated by running multiple initializations.

### 3.2. Supervised Learning Algorithms

Supervised learning uses labeled datasets—where each sample is associated with a known outcome—to train a model that can predict the label of new, unseen samples. This paradigm is central to disease diagnosis, where the goal is to classify patient samples as healthy or diseased or to quantify biomarker concentrations. Supervised tasks are further divided into classification (discrete outputs) and regression (continuous outputs) [50].

Classification algorithms predict categorical outcomes. SVM is a powerful binary classifier that finds the optimal hyperplane maximizing the margin between two classes [32,51]. By employing kernel functions (e.g., linear, polynomial, or radial basis function), SVM can handle non-linear separability. In optical sensing, SVM has been widely used to distinguish between different cancer types based on SERS spectra, obtaining high accuracy even with limited sample sizes. Its robustness to overfitting and ability to work with high-dimensional data make it a preferred choice for many diagnostic applications. However, SVM struggles with multi-class problems and large-scale datasets, and its performance heavily depends on kernel selection and parameter tuning.

RF is an ensemble learning method that constructs a multitude of decision trees during training and outputs the majority vote (for classification) or average (for regression) of individual trees. RF is inherently resistant to overfitting, handles missing values well, and provides feature importance rankings, which are valuable for identifying key biomarkers [52,53]. In colorimetric sensor arrays for urinary tract infection diagnosis, RF combined with other algorithms yielded near-perfect accuracy. Nevertheless, RF models can be less interpretable and require longer training times for complex datasets.

ANNs are inspired by biological neural systems and consist of interconnected layers of neurons (input, hidden, and output). ANNs can approximate any non-linear function given sufficient data and hidden units. They are particularly effective for processing noisy, high-dimensional sensor data because they automatically extract relevant features through hierarchical transformations [18,54,55]. For instance, ANNs have been used to analyze fluorescence patterns from exosome-based sensors for breast cancer subtyping. The major limitations include the need for large training datasets, sensitivity to hyperparameter choices (e.g., learning rate, number of layers), and the risk of overfitting, which can be mitigated by regularization and cross-validation.

Other classification algorithms, such as k-Nearest Neighbors (k-NNs) and Naive Bayes, are also frequently applied due to their simplicity and interpretability, though they often underperform compared to ensemble or deep learning methods on complex sensor data [56].

Regression algorithms predict continuous values, such as analyte concentration or disease severity. Multiple Linear Regression (MLR) models linear relationships between input features and the target variable. While simple and interpretable, MLR fails to capture non-linear interactions and is sensitive to multicollinearity, limiting its utility in complex biological systems.

Random Forest Regression extends the RF framework to continuous prediction tasks, offering robust performance and feature importance insights. It has been successfully employed to quantify biomarker levels from electrochemical or optical signals, attaining low prediction errors and high correlation with reference methods. Gradient Boosting algorithms (e.g., XGBoost and LightGBM) are another powerful family of ensemble methods that sequentially correct errors of previous models, often yielding state-of-the-art results in both classification and regression tasks for biosensor data.

### 3.3. Deep Learning Algorithms

Deep learning is a specialized subfield of ML that employs multi-layer neural networks (deep neural networks) to automatically learn hierarchical representations from raw data. Unlike classical ML algorithms that rely on handcrafted features, deep learning models can process unstructured data such as images, spectra, and time series directly, making them particularly suitable for analyzing complex optical sensor outputs [57].

Convolutional Neural Networks (CNNs) are the most widely used deep architecture for grid-like data, such as 2D spectral images or colorimetric array photographs. CNNs leverage convolutional layers to detect local patterns (e.g., edges, peaks) and pooling layers to demonstrate translation invariance, followed by fully connected layers for classification. In SERS-based diagnostics, CNNs have been trained on raw spectral data to distinguish liver diseases with >97% accuracy, outperforming traditional classifiers. For instance, Cheng et al. integrated a SERS biosensor comprising Au-Ag nanocomplex-decorated ZnO nanopillars with a custom CNN classifier, reporting 97.78% accuracy in distinguishing normal controls, hepatocellular carcinoma, and hepatitis B patients from serum samples within 1 min [58]. Similarly, Xiong et al. developed a label-free serum SERS platform combined with a 1D-CNN, attaining 98.27% accuracy in classifying healthy controls and three different cancers (bladder cancer, adrenal cancer, and acute myeloid leukemia), surpassing PCA-LDA (90.91%), PCA-SVM (90.45%), and classical deep learning models including AlexNet (85.36%) and VGGNet (93.36%) [59].

Recurrent Neural Networks (RNNs), particularly Long Short-Term Memory (LSTM) networks, are designed for sequential data and are adept at capturing temporal dependencies [60]. In wearable biosensors or dynamic assays, LSTM models can predict hormone fluctuations, glucose trends, or vital sign anomalies from time-series signals. For example, LSTM has been used to estimate analyte concentrations from electrochemical aptamer sensors, reaching >90% accuracy after data augmentation.

More recently, architectures such as Kolmogorov-Arnold Networks (KAN) have been proposed as alternatives to traditional MLPs, replacing fixed activation functions with learnable spline-based functions [15,35]. While KAN theoretically offers better approximation capabilities, its practical application in biosensing remains limited due to overfitting risks with small datasets.

### 3.4. Performance Evaluation Metrics for ML Models

In disease diagnosis applications, rigorous evaluation of ML model performance is essential to ensure clinical reliability and translational value. Several standardized metrics have been established to comprehensively assess diagnostic models, each providing unique insights into model capabilities and limitations [61,62]. The confusion matrix serves as the foundation for performance evaluation, organizing predictions into four categories: true positives (TP), true negatives (TN), false positives (FP), and false negatives (FN). From this matrix, multiple critical metrics are derived.

Accuracy, defined as (TP + TN)/(TP + TN + FP + FN), represents the proportion of correctly classified samples among all predictions. Although intuitive, accuracy can be misleading when datasets are imbalanced [63,64]. In some studies, optical sensing arrays have demonstrated exceptionally high classification accuracy in controlled laboratory settings. For example, an AuNP-based colorimetric array demonstrated 100% accuracy in discriminating 17 microbial species at the genus level [65], while a fluorescent nanozyme array attained 100% accuracy in distinguishing five cancer cell-derived exosomes and in predicting 28 unknown samples via Linear Discriminant Analysis (LDA)-based discriminant analysis [66]. However, these values should be interpreted with caution, as they were derived from limited sample sizes (typically 5–6 replicates per group) and validated primarily through cross-validation or small test sets rather than independent, large-scale external cohorts. 

Notably, Cheng et al. engineered a nanoplasmonics biosensing chip (NBC) consisting of AgNP-decorated ZnO nanorods on cellulose filter paper for label-free serum SERS detection of hepatocellular carcinoma (HCC). A deep neural network (DNN) classifier trained on 1140 serum SERS spectra secured 91% accuracy on an external validation set, with four-fold cross-validation accuracies of 97.3–98.6% and 100% sensitivity/specificity for HCC detection [67].

Sensitivity (also called recall or true positive rate), calculated as TP/(TP + FN), measures the model’s ability to correctly identify diseased individuals. This metric is particularly critical for acute infectious diseases, where missing a positive case can lead to delayed treatment and further transmission. Among the studies summarized in Table 1, sensitivity was not reported for most colorimetric and fluorescent sensors, but label-free SERS for neuropsychiatric systemic lupus erythematosus (NPSLE) reported a sensitivity of 95.9% [68], demonstrating excellent true-positive detection capability. Specificity, defined as TN/(TN + FP), evaluates the capacity to correctly identify non-diseased individuals. High specificity is essential to minimize false positives, which can cause unnecessary patient anxiety and additional healthcare costs. The same NPSLE study reported a specificity of 93.5% [68], indicating a well-balanced performance.

Beyond these core metrics, the area under the receiver operating characteristic curve (AUC) and the F1 score are also widely used, though they are not uniformly reported across the studies in Table 1. The receiver operating characteristic (ROC) curve plots the true positive rate against the false positive rate across all thresholds, and AUC quantifies overall discriminatory power. While many studies focused on accuracy, a few also provided sensitivity and specificity (e.g., the NPSLE case). For SERS-based Alzheimer’s disease diagnosis, CNN models yielded 92% accuracy [72]. In the microfluidic optical category, a portable sensor integrating CatBoost and RF algorithms obtained 92% accuracy for multiplex detection of cardiac biomarkers (cTnI, HDL, and LDL) in acute myocardial infarction diagnosis [75], while another K-NN-assisted microfluidic platform demonstrated 96% accuracy for Mycobacterium tuberculosis detection, highlighting the versatility of these miniaturized systems for infectious disease diagnostics. Additionally, a wearable physiological sensor employing XGBoost realized 96.12% accuracy for real-time stress detection [76], showcasing the potential of ML-enhanced wearable platforms for continuous health monitoring—though external factors such as motion artifacts and individual variability remain significant challenges. Collectively, the representative metrics in Table 1 illustrate that ML-assisted optical sensing arrays can deliver high diagnostic accuracies (often >90%), but the lack of standardized reporting for sensitivity, specificity, and AUC in many studies highlights the urgent need for more comprehensive evaluation protocols to facilitate cross-study comparison and accelerate clinical translation.

## 4. Application of ML in Optical Sensing Arrays for Disease Diagnosis

Optical sensing arrays generate multidimensional optical fingerprints upon interaction with target analytes. These fingerprints—encompassing absorbance, fluorescence intensity, or Raman signals—are subsequently processed by ML algorithms to determine the biochemical composition of clinical samples. In this section, we systematically review representative applications of ML-assisted optical sensing arrays in disease diagnosis.

### 4.1. Colorimetric Sensor Arrays

Colorimetric sensor arrays transduce molecular recognition events into measurable color changes via chemical reactions or physical adsorption. These changes can be quantified using UV-Vis spectrophotometers or microplate readers, and with the assistance of ML algorithms, precise disease diagnosis can be attained. Based on the nature of the sensing elements, colorimetric sensor arrays can be broadly classified into chromogenic reagent-assisted sensors and nanomaterial-based optical sensors.

#### 4.1.1. Chromogenic Reagent-Assisted Sensors

Nanozymes, nanomaterials with enzyme-like catalytic properties, offer advantages including high stability, facile functionalization, and low cost, making them excellent sensing units for colorimetric sensor arrays [28,78,79]. Among these, carbon dots (CDs) have garnered significant attention in biochemical sensing due to their superior physicochemical properties and excellent biocompatibility. Metal ions, with their unique electronic structures and superior catalytic activity, can significantly enhance the enzyme-like activity of CDs.

**Figure 3:**
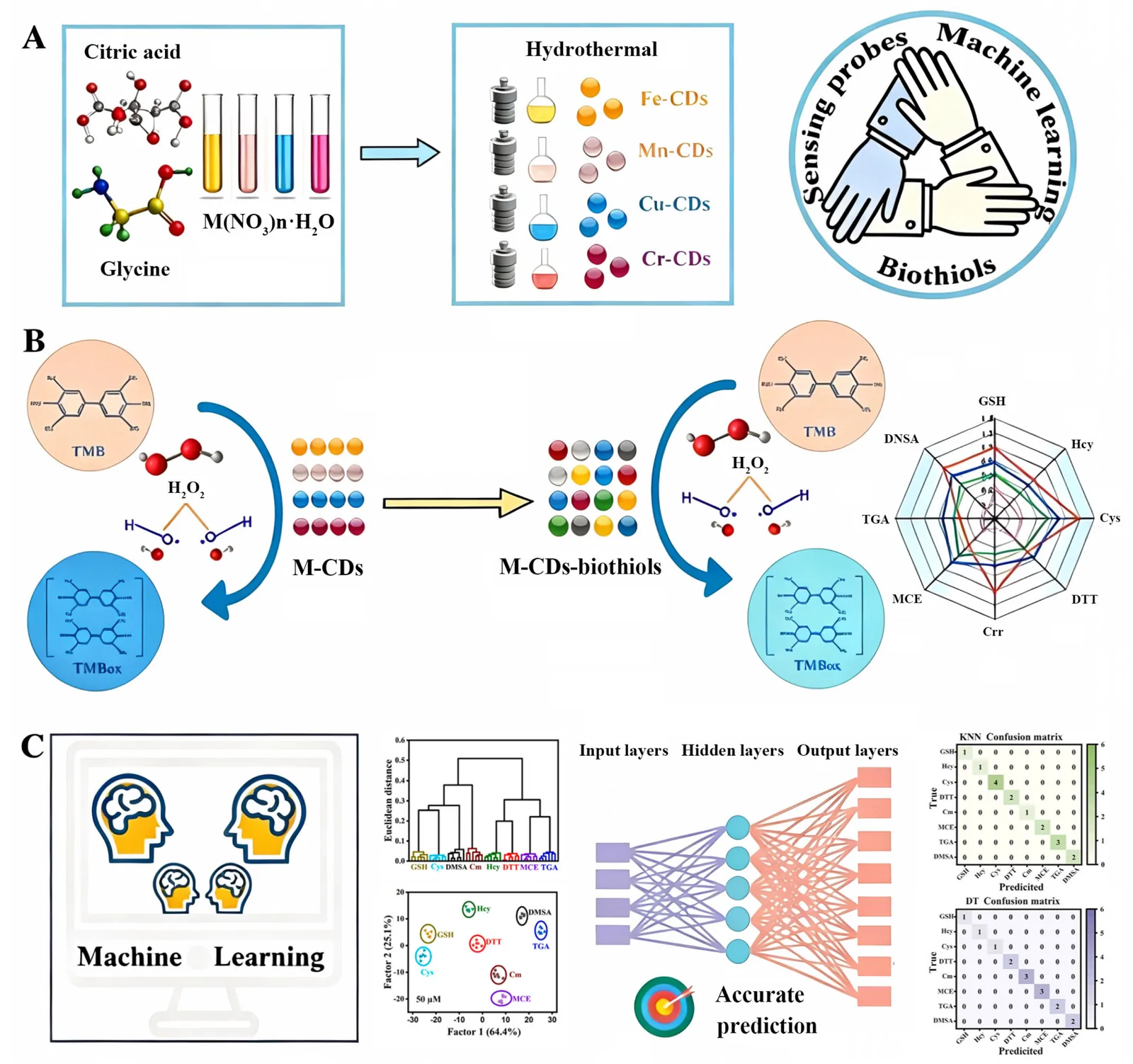
(**A**) Schematic illustration of a colorimetric sensor array based on M−CDs nanozymes for the discrimination of biothiols. (**B**) Preparation process of different M−CDs. (**C**) Discrimination and prediction of biothiols with the assistance of ML algorithms (reprinted with permission from Ref. [80]. Copyright 2025, Elsevier).

Mingming Wei et al. synthesized four types of metal-doped carbon dots (M-CDs)—Fe-CDs, Mn-CDs, Cu-CDs, and Cr-CDs—via a one-pot hydrothermal method using citric acid, ethylenediamine, and corresponding metal salts as precursors [80]. These nanozymes exhibited peroxidase-like activity and were applied for biothiol detection. The M-CDs-based colorimetric sensor array, in combination with multiple ML algorithms (LDA, HCA, ANN, K-NN, DT, and SVM), successfully discriminated eight biothiols with a detection limit of 40 nM and further reached accurate identification of tumor cells and bacteria through pattern recognition (Figure 3). It should be noted, however, that the reported promising classification accuracy was derived from a dataset with only six replicate measurements per concentration and per biothiol species; the absence of an independent external validation cohort raises concerns regarding potential overfitting, although the study partially addressed this through blind testing of 25 unknown samples. Direct comparisons to conventional non-ML analytical methods—such as HPLC or GC-MS—were not systematically provided, making it difficult to quantify the true advantage of the ML integration over established laboratory techniques. Additionally, the SVM algorithm alone achieved only 80% accuracy on the test set, whereas the LDA-SVM cascade model improved this to 100%, highlighting that algorithmic selection and optimization influence reported performance.

Single-atom nanozymes represent a recent significant advancement in this field. These materials feature isolated metal atoms as catalytic centers, maximizing atomic utilization efficiency and enhancing detection performance [51]. Fang Xin Hu et al. constructed an iron single-atom nanozyme-based colorimetric sensor array for urinary tract infection (UTI) diagnosis [19]. By assembling four recognition ligands (antibiotics, boric acid, CTAB, and D-alanine) on Fe single-atom catalysts and combining this with ML algorithms, they reported 97% diagnostic accuracy across 60 clinical samples. The portability of this sensor platform makes it suitable for intelligent home-based diagnostics.

#### 4.1.2. Nanomaterial-Based Optical Sensors

Gold nanoparticles (AuNPs) exhibit unique size-dependent optical properties, high surface plasmon resonance (SPR) effects, excellent biocompatibility, and facile surface modification. AuNPs display distinct colors depending on their size and shape. The binding of target molecules to functionalized AuNP surfaces induces aggregation or dispersion, resulting in color changes within the sensing system. By analyzing the degree of color change, both qualitative and quantitative analyses of target molecules can be performed [11,69,81].

Antibiotic-resistant pathogens pose a major challenge to public health security. Current detection methods for bacterial resistance, including antibiotic susceptibility testing and nucleic acid amplification, often require extended detection times or large-scale precision instruments, limiting their utility in clinical acute infectious disease diagnosis. To address this, Xianyu et al. devised an AuNP-based colorimetric sensor array for detecting antibiotic resistance in ESKAPE pathogens, demonstrating rapid and precise diagnosis within 20 min with 89.7% accuracy [69].

Furthermore, microorganisms can synthesize AuNPs using their own enzymes, proteins, lipids, and carbohydrates. Since different microorganisms contain varying types and amounts of bioactive molecules, the AuNPs produced exhibit distinct colors and morphological characteristics, providing a theoretical basis for precise pathogen identification. Ting Yu et al. proposed an ML-assisted biosensing strategy that converts pathogen detection into AuNP characterization. Using 17 common microorganisms as target analytes for AuNP synthesis, they measured UV-Vis absorption spectra, particle sizes, and surface charges [82]. ML algorithms yielded classification accuracy exceeding 99.5% for more than ten microbial taxa across taxonomic levels—including phylum, family, genus, and species—demonstrating performance comparable to that of gold-standard 16S rRNA gene sequencing. These results underscore the considerable potential of ML-based approaches in rapid pathogen identification and clinical infectious disease diagnosis [38,83,84,85].

### 4.2. Fluorescent Sensor Arrays

Fluorescent sensor arrays rely on the specific interactions between fluorescent probes and target molecules, which alter the microenvironment of fluorophores and consequently change fluorescence intensity, lifetime, or polarization parameters. These fluorescence variations are closely correlated with analyte types and concentrations, and ML algorithms can analyze the resulting multidimensional data to establish diagnostic patterns. Based on the types of disease biomarkers targeted, fluorescent sensor arrays can be further categorized into nucleic acid sensors, exosome sensors, and microbial sensors, each with distinct probe designs and ML-assisted analytical workflows.

#### 4.2.1. Nucleic Acid Sensors

**Figure 4:**
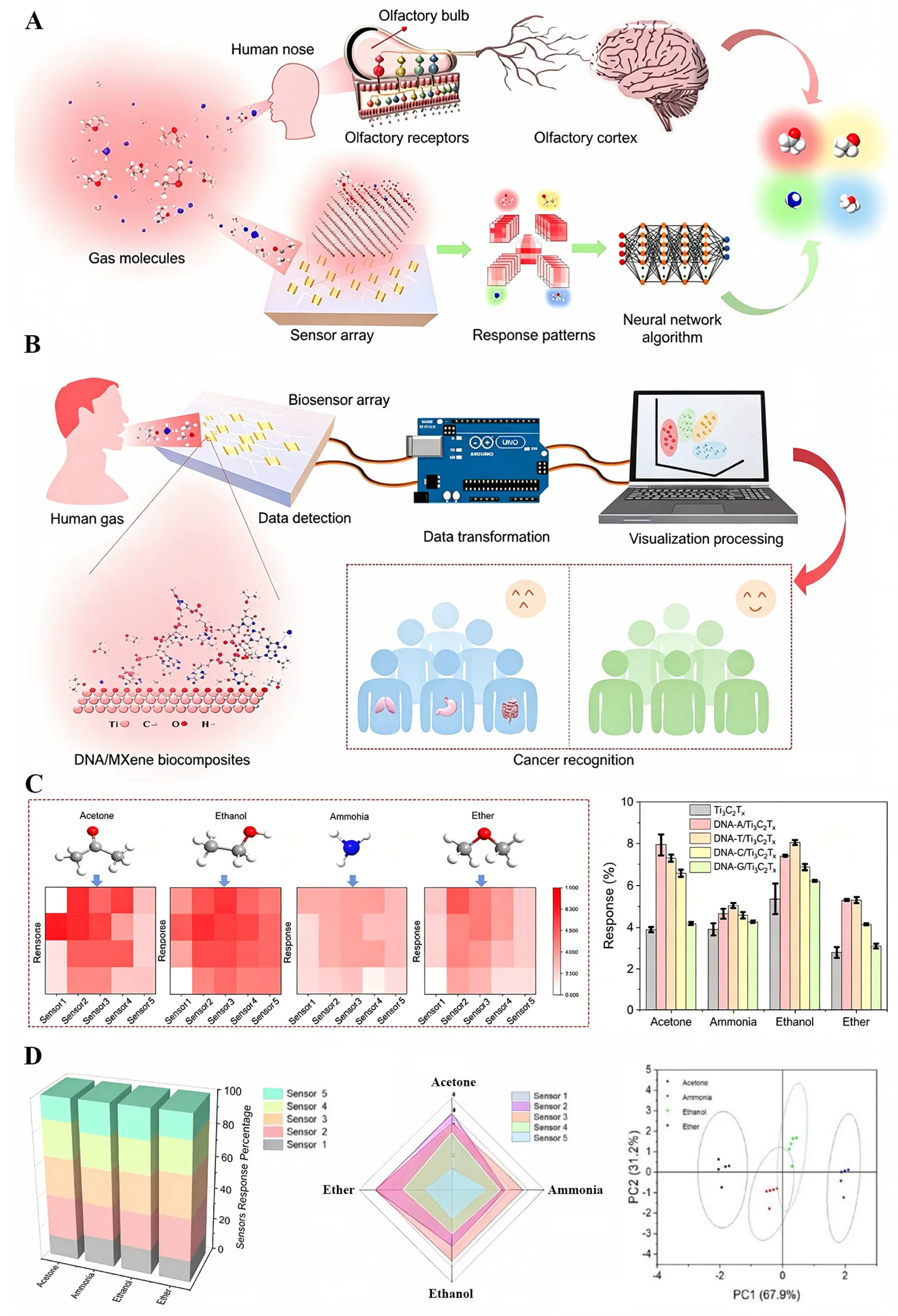
(**A**) Schematic diagram of the human olfactory system and the sensor array system for gas identification. (**B**) Schematic diagram of the ITP platform for cancer recognition with the assistance of ML. (**C**) Sensing response of the 5C−BBA for four typical gases at a concentration of 20 ppm. (**D**) Percentage of the response of each sensor in the 5C−BBA to the detection of a single gas (reprinted with permission from Ref. [22]. Copyright 2025, American Chemical Society).

Nucleic acids serve as critical biomarkers for identifying pathogens, genetic diseases, and cancers. In acute infectious disease diagnosis, viral or bacterial nucleic acid sequences provide direct evidence of pathogen presence [48,86]. Accurate differentiation of infection types enables personalized treatment and reduces inappropriate antibiotic use. Shanshan Liu et al. fabricated a bioinspired biosensor array (BBA) based on DNA/MXene composites for noninvasive cancer recognition via exhaled breath detection [22]. The diagnostic workflow comprises three steps: first, screening five DNA/MXene composites with excellent sensing properties through orthogonal experiments and combining them with pure MXene to construct a Six-Channel Bioinspired Biosensor Array (6C-BBA); second, integrating the 6C-BBA with an Arduino-Based Instant Testing Platform (ITP) and multiple ML algorithms (LR, LDA, K-NN, CART, NB, and SVM) for gas recognition; and third, validating the system on fruit odor molecules and exhaled breath samples from stomach cancer, lung cancer, colorectal cancer patients, and healthy individuals, realizing recognition accuracies of 86.3%, 94.1%, 89.5%, and 86.3%, respectively (Figure 4). Similarly, while these results are promising, the validation was performed on a relatively small patient cohort using cross-validation rather than independent external testing; the limited sample size may affect the statistical power of the reported accuracies.

Linlin Yang et al. further integrated the DNA molecular computing system into a spatially localized DNA origami framework, constructing a DNA molecular classifier for lung cancer diagnosis [70]. By performing multiplication, addition, and subtraction operations on nucleic acid inputs—simulating neuronal computation—they reduced computational complexity. The system secured 90% accuracy in distinguishing non-small cell lung cancer from healthy samples.

#### 4.2.2. Exosome Sensors

**Figure 5:**
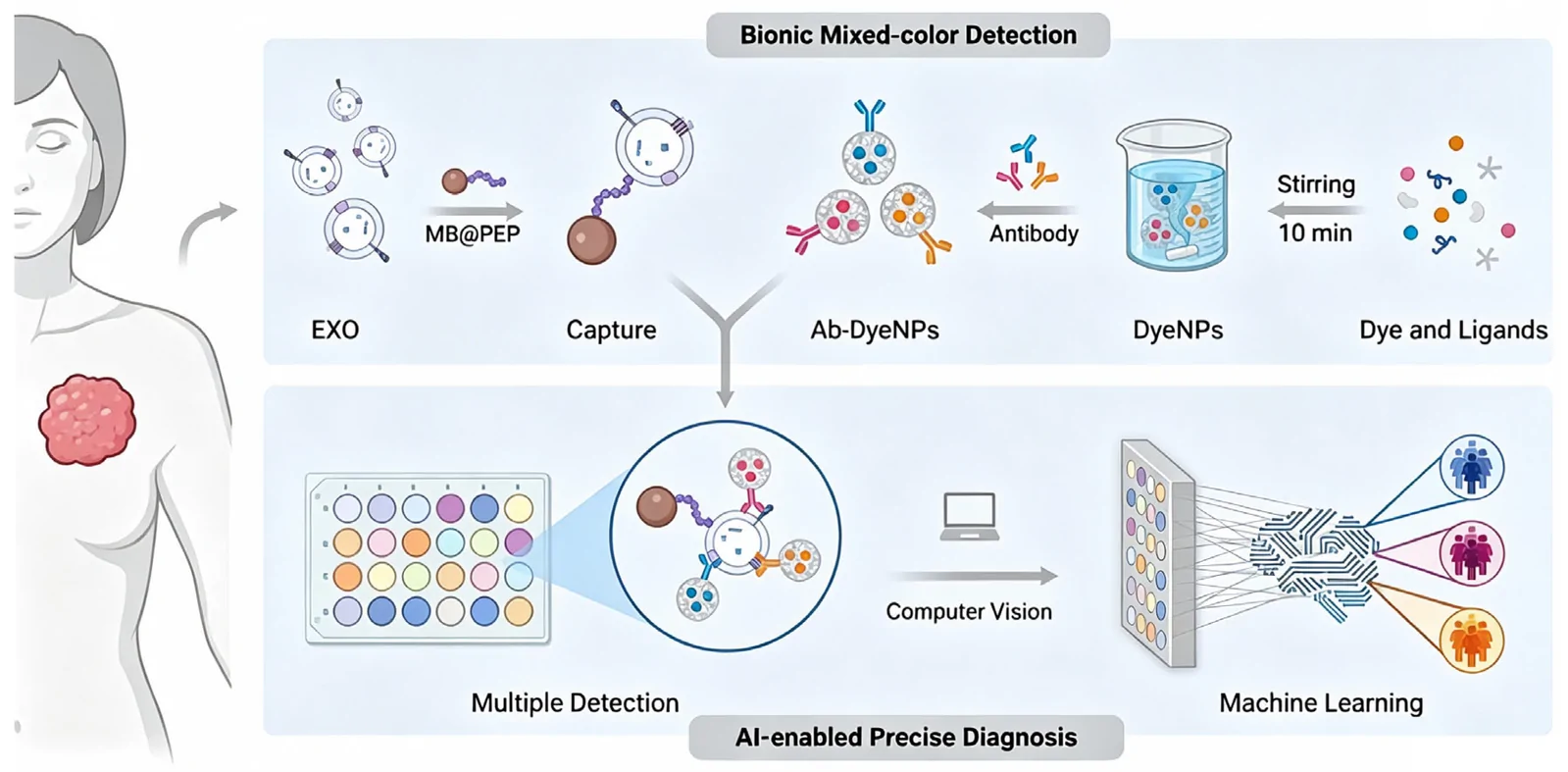
Scheme of AI-enabled colorimetric analysis of EXO for precise diagnosis of breast cancer (reprinted with permission from Ref. [89]. Copyright 2026, Royal Society of Chemistry).

Exosomes are small vesicles (30–150 nm in diameter) secreted by cells, containing proteins, RNA, and lipids that reflect physiological or pathological changes, making them valuable for disease diagnosis [87,88]. The application of AI-assisted colorimetric sensing to exosome detection is illustrated in Figure 5. Inspired by the human visual system, which perceives myriad colors using only three types of cone cells, Xinyu Qu et al. established an AI-enabled bionic mixed-color sensing technology for breast cancer diagnosis through exosome analysis [89]. This approach gives exosomes a unique “mixed color fingerprint” by simultaneously binding three color-coded nanoprobes (Ab-DyeNPs) that target distinct surface proteins—PD-L1, EpCAM, and HER-2. The resulting mixed-color signal is captured by computer vision and subsequently processed by ML algorithms to generate a diagnostic conclusion. For cell line models, this technology accurately differentiated various breast cancer subtypes with up to 100% accuracy. More importantly, when evaluated on 60 clinical serum samples, it attained 96.7% accuracy in subtyping breast cancer, outperforming both single-target and conventional multi-target analysis strategies.

Jinxiu Wei et al. introduced a super-resolution tricolor fluorescence co-localization (SR-TFC) method to address nonspecific adsorption in exosome immunoassays. Exosomes, capture probes, and recognition probes were labeled with three distinct fluorescent dyes [71]. For specific immune binding sites, the three probes spatially overlapped (appearing white), whereas nonspecific sites showed no overlap. A pixel-counting algorithm (CFPP) eliminated nonspecific binding at the single-pixel level. By profiling PD-L1, HER2, and EpCAM on breast cancer exosomes, this strategy accurately classified different breast cancer subtypes and clinical blood samples, highlighting its translational promise in clinical diagnostics.

#### 4.2.3. Microbial Sensors

Microorganisms as biomarkers are playing increasingly important roles in clinical diagnosis [82,85,90]. Analyzing microbial community composition can provide early diagnosis and treatment monitoring for infectious diseases, immune disorders, and chronic conditions. Yang Yu et al. fabricated a fluorescent sensor array using negatively charged poly(p-aryleneethynylene) and six positively charged aggregation-induced emission (AIE) dye electrostatic complexes for clinical UTI bacterial diagnosis, reaching rapid identification of different UTI clinical samples through multiple ML algorithms [91]. To enhance the selectivity of array-based sensors toward bacteria, Xin Wang et al. designed a metabolic labeling-assisted fluorescence sensor for pathogen antibiotic resistance analysis [84]. Using dibenzocyclooctyne-functionalized upconversion nanoparticles (UCNPs) as sensing units, pathogens under different antibiotic conditions were metabolically labeled with 3-azido-D-alanine, followed by click reactions with UCNPs to generate fluorescence signals. LDA and UMAP algorithms processed these signals for pathogen identification in complex systems, considerably improving the selectivity of the sensor array toward pathogenic microorganisms.

### 4.3. SERS Sensor Arrays

SERS sensors combine high sensitivity with molecular fingerprinting capabilities, making them particularly valuable for biomarker detection [24,92,93]. ML algorithms are especially effective for processing complex SERS spectral data, thereby allowing automated feature extraction and pattern recognition from high-dimensional Raman signatures. Based on their detection strategies, SERS sensors are classified into label-free and labeled types. The following subsections review representative applications of each type, with particular attention to ML model architectures (e.g., CNNs, ANNs) and their role in enhancing diagnostic sensitivity and specificity in complex biological matrices [94,95].

#### 4.3.1. Label-Free SERS Sensors

**Figure 6:**
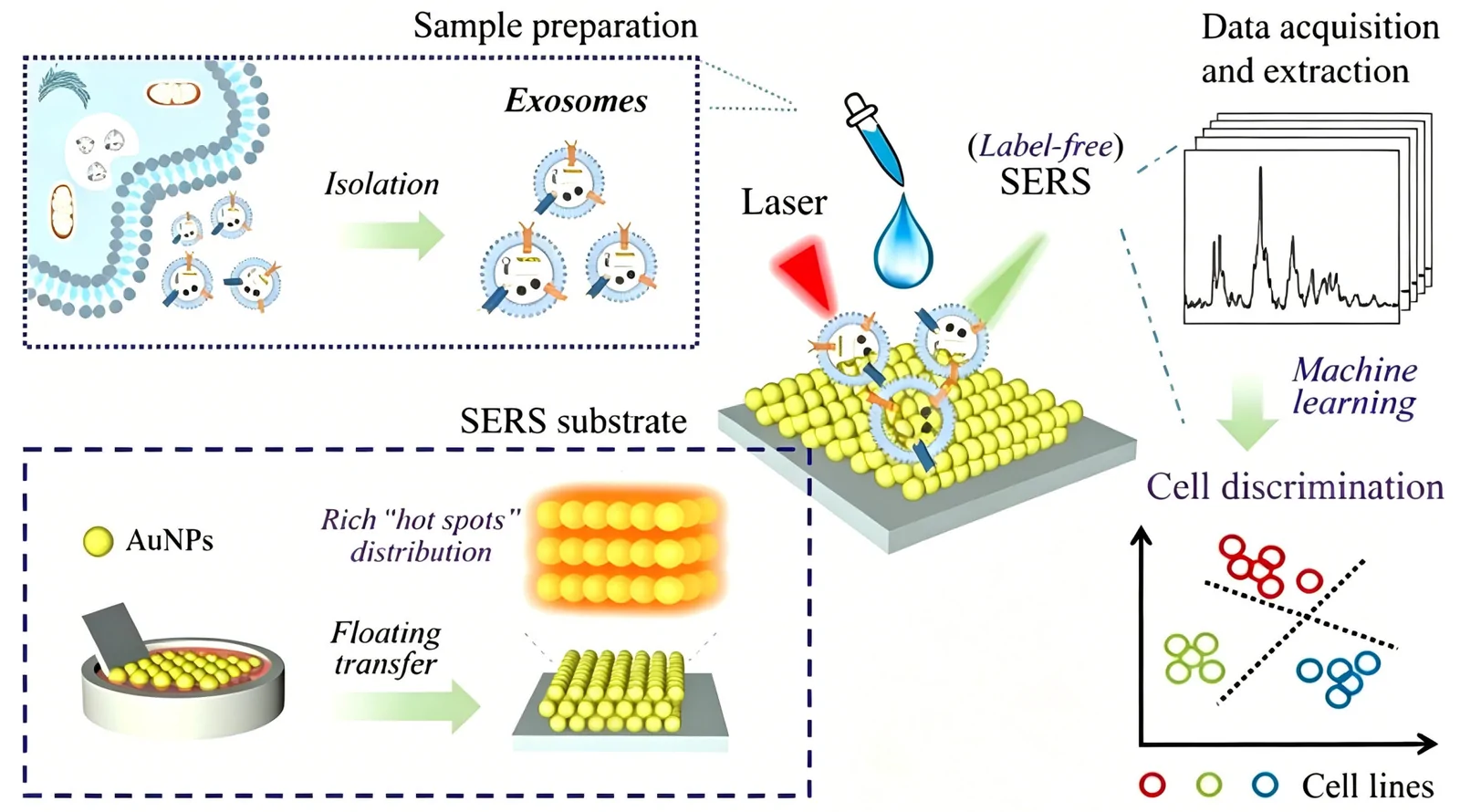
Schematic diagram of label-free SERS detection and accurate fuzzy discrimination of exosomes derived from different cell lines using trilayered plasmonic AuNP nanomembranes as 3D SERS substrates combined with ML-based signal acquisition and extraction (reprinted with permission from Ref. [96]. Copyright 2023, American Chemical Society).

Label-free SERS sensors require no additional label molecules and directly utilize SERS signals generated from the interaction between target molecules and metal surfaces, offering simple operation and low cost. Diao et al. developed an ML-based label-free SERS platform for exosome profiling, utilizing three-dimensional plasmonic gold nanofilms as SERS substrates to directly acquire fingerprint spectra from exosomes without any labeling [96], as shown in Figure 6. Combined with ML algorithms, this platform enabled accurate fuzzy diagnosis of breast and cervical cancers, as well as dynamic monitoring of drug therapeutic processes. In nucleic acid detection, conventional SERS sensors relying on hybridization analysis often suffer from poor reproducibility and low signal intensity. To address this, Hyungi Kim et al. constructed a deep learning-assisted ZnO-AuNP SERS substrate detection system for rapid and sensitive nucleic acid diagnostics, achieving 100% sensitivity and specificity while reducing analysis time to within 20 min [32]. Label-free SERS sensors have also been widely applied in disease subtyping. For example, Mi et al. developed a label-free serum microsphere-coupled SERS platform combined with ML-assisted analysis for the diagnosis of neuropsychiatric systemic lupus erythematosus (NPSLE), attaining high diagnostic accuracy [68].

#### 4.3.2. Labeled SERS Sensors

**Figure 7:**
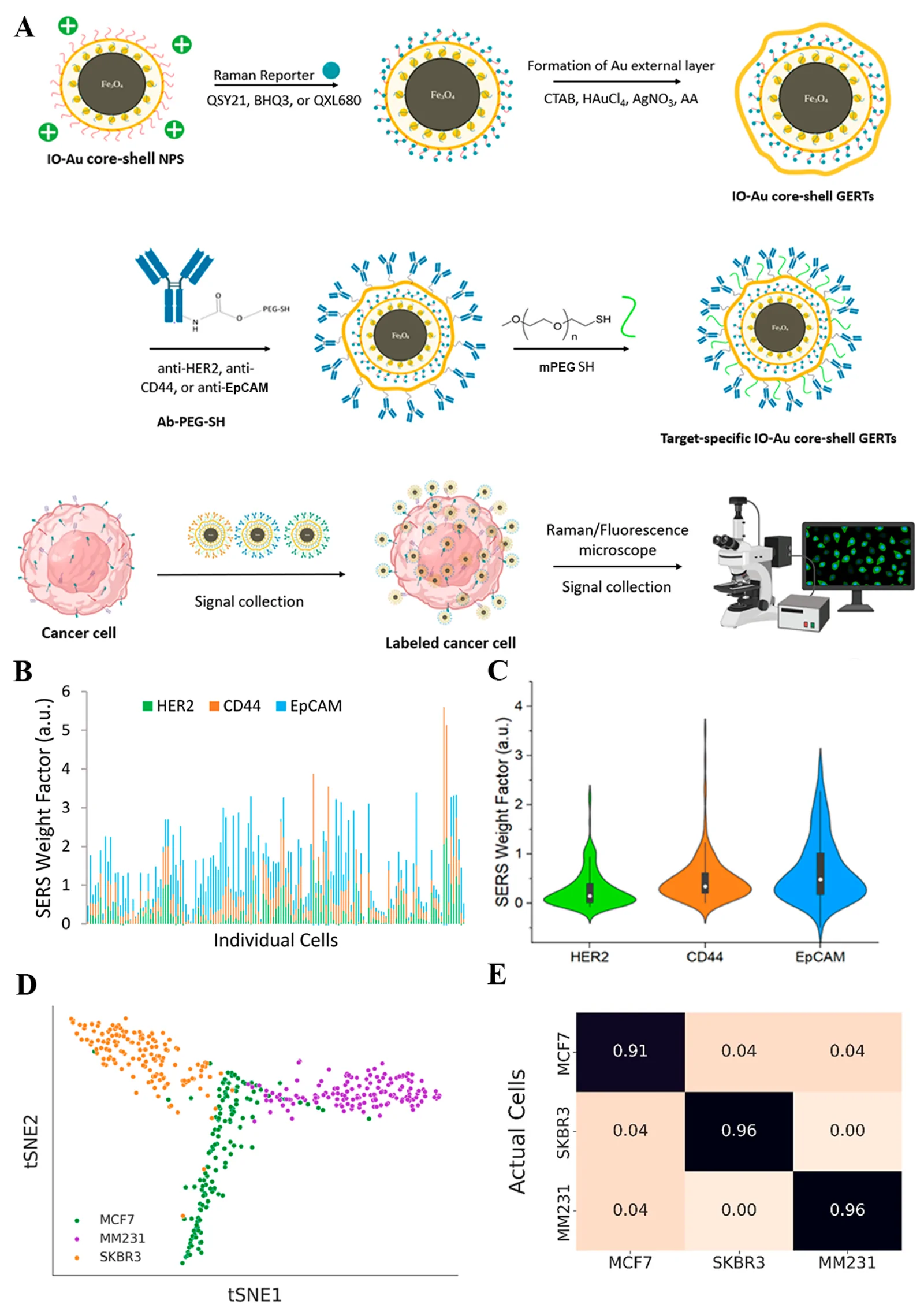
(**A**) Schematic of the synthesis and conjugation of multicolor IO/Au/GERTs. Three different cancer surface markers were targeted with their specific antibodies and Raman reporters (QSY21, BHQ3, or QXL680). (**B**) SERS weight factors for each protein marker from 150 SKBR3, MM231, and MCF7 mixed cells. (**C**) Violin plots showing the distribution of each cancer marker on single cells. (**D**) t-SNE visualization of the labeled training data. (**E**) Confusion matrix from the training data with the actual cells along the *y*-axis and the predicted cells along the *x*-axis. Values are presented as fractions (reprinted with permission from Ref. [44]. Copyright 2024, American Chemical Society).

Labeled SERS sensors adsorb specific biomolecules onto metal nanostructure surfaces, generating strong Raman scattering signals upon laser excitation for highly sensitive biochemical detection. For instance, Rodriguez-Nieves et al. established a labeled SERS platform based on magnetic core-gold shell gap-enhanced Raman tags (GERTs) for multiplexed detection and classification of breast cancer surface proteins [44], as shown in Figure 7. Three distinct Raman reporters were encapsulated within the gap between the inner and outer gold shells, which not only prevented reporter leakage and photobleaching but also generated strong electromagnetic field enhancement through plasmonic coupling. Combined with a random forest ML classifier, the method achieved a profiling and classification accuracy of 93.9% for three breast cancer cell lines.

Antibodies are widely employed as recognition elements in biosensing due to their high affinity and specificity. However, they suffer from inherent limitations, including stringent storage conditions, high production costs, loss of biological activity, batch-to-batch variability, and potential ethical concerns [97,98]. These drawbacks restrict their application in extreme environments, low-cost screening tests, and small molecule analysis, highlighting the need for highly stable, low-cost non-protein recognition alternatives. In response to these challenges, various synthetic alternatives have been devised, including aptamers and molecularly imprinted polymers (MIPs), which offer advantages such as thermal stability, long shelf life, cost-efficient chemical synthesis, and negligible batch-to-batch variations [99,100]. Additionally, extracellular matrix components serve as biomarkers for pathogen identification. Leong et al. formulated a SERS-based surface chemotaxonomy method for bacterial identification by leveraging the distinct chemical signatures of bacterial extracellular matrices [74]. This approach demonstrated over 98% recognition accuracy across six bacterial species without requiring gene amplification, biochemical testing, or specific biomarker recognition, pointing to broad diagnostic applicability for rapid and universal pathogen identification.

### 4.4. Comparative Analysis of Modalities

To guide the rational selection of sensing platforms and corresponding ML strategies for specific diagnostic tasks, Table 2 presents a direct and comprehensive cross-modality comparison of colorimetric, fluorescent, and SERS sensor arrays. This comparison systematically evaluates each modality across multiple critical dimensions, including typical limit of detection, cost per test, instrumentation and sample preparation requirements, assay time, suitable application scenarios, data characteristics, and commonly employed ML algorithms. As indicated, colorimetric arrays offer the simplest operation and lowest cost, generating static RGB or absorbance data that are effectively handled by classical supervised classifiers (e.g., SVM, RF) after simple feature extraction, making them ideal for point-of-care and smartphone-based testing. Fluorescent arrays provide higher sensitivity and multiplexing capability yet require more complex probe design and optical instrumentation; their time-resolved or ratiometric signals often benefit from ANNs or deep learning when sufficiently large datasets are available. Conversely, SERS arrays deliver the highest molecular specificity and fingerprinting capability at the expense of higher substrate fabrication costs and spectral variability; their high-dimensional spectral data are particularly amenable to deep learning architectures (CNNs, 1D-CNNs) and advanced feature engineering. This structured comparison underscores that the optimal ML pipeline is highly modality-dependent and should be selected based on data characteristics, clinical requirements, and available resources, thereby fulfilling the critical evaluation framework promised in the abstract.

## 5. Challenges and Future Prospects

Despite the considerable progress obtained in ML-assisted optical sensing arrays for disease diagnosis, several critical challenges persist across the entire workflow—from sensor fabrication and data acquisition to model deployment and clinical translation. These challenges are inherently interconnected, and addressing them requires a holistic approach that integrates advances in materials science, data engineering, algorithm design, and regulatory science.

### 5.1. Data Quality and Standardization for Optical Sensor Arrays

The performance of ML models is fundamentally constrained by the quality, quantity, and representativeness of training data [101,102]. Optical sensing arrays generate high-dimensional, multiparametric datasets that are often plagued by noise, batch effects, and missing values, complicating the extraction of reliable diagnostic signatures [4,103].

Array-Specific Data Challenges: Unlike many other biomedical data types, optical sensor arrays face unique data quality issues. Batch-to-batch variations in nanoparticle synthesis, substrate fabrication, and probe functionalization can introduce systematic spectral shifts or intensity fluctuations that obscure biologically meaningful signals. Environmental factors—including temperature, humidity, and ambient light—can affect colorimetric readouts and fluorescence stability, particularly for point-of-care applications. Additionally, clinical samples such as serum, urine, or saliva contain complex matrices that cause nonspecific adsorption or quenching, leading to inconsistent sensor responses across different sample types.

Data Scarcity and Imbalance: Acquiring large-scale, well-annotated clinical datasets remains a formidable challenge, particularly for rare diseases or early-stage lesions where patient samples are inherently limited. Moreover, the class distribution in real-world clinical settings is often skewed, with certain disease subtypes or demographic groups underrepresented [104]. Such imbalances can bias ML models toward majority classes, leading to poor generalization and reduced diagnostic sensitivity for minority populations.

Mitigation Strategies: Several complementary approaches have been proposed to address these data-related challenges. Transfer learning, which involves pre-training models on large public spectral libraries or related datasets, followed by fine-tuning on limited clinical data, has shown promise in improving generalization with small sample sizes [95,105]. For batch effect correction, ML-based normalization algorithms—such as ComBat and its variants—have been adapted from genomics to optical sensing data, reducing cross-batch variability and enhancing cross-institutional reproducibility. Standardized data acquisition protocols, reference materials, and centralized data repositories are essential to facilitate multi-center collaboration and ensure comparability across studies [106].

### 5.2. Model Robustness and Generalization Across Sensor Platforms

While optical sensing arrays achieve high diagnostic accuracy on training cohorts, their performance often declines when applied to new patient populations, clinical settings, or sensor platforms. This generalization gap remains one of the most significant barriers to clinical adoption. Two complementary strategies are critical to bridging this gap: improving sensing interface design and leveraging multimodal data integration [50,55,92].

Sensor-Specific Generalization Issues: Models trained on data from one sensor batch or fabrication method may fail when applied to another due to subtle differences in nanoparticle size distribution, surface coverage, or substrate morphology. This is particularly problematic for SERS substrates, where even minor variations in nanostructure geometry can significantly alter spectral profiles. Similarly, colorimetric arrays using different batches of chromogenic reagents or nanozymes may exhibit different dynamic ranges and sensitivity thresholds.

Strategies for Improving Robustness: Domain adaptation techniques, including unsupervised adversarial training and supervised domain alignment, offer potential solutions for cross-institutional and cross-platform deployment by learning domain-invariant feature representations. Rigorous validation strategies—including k-fold cross-validation, leave-one-batch-out validation, and independent external validation cohorts—provide increasingly stringent tests of model performance. Calibration analysis, which assesses the alignment between predicted probabilities and observed outcomes, is critical for clinical decision-making, as poorly calibrated models can produce overconfident predictions that undermine clinician trust.

Sensing Interface Optimization: The robustness of ML-assisted optical arrays can also be improved by modulating the interfacial properties of sensor elements. Rational design of recognition ligands—including aptamers, molecularly imprinted polymers, and peptide-based receptors—can enhance target binding affinity and selectivity in complex biological matrices [107]. Surface chemistry modifications that resist nonspecific adsorption (e.g., zwitterionic coatings, polyethylene glycol brushes) are critical for maintaining signal integrity across different clinical sample types [97,108,109].

### 5.3. Model Interpretability for Array-Based Diagnostics

The inherent “black box” nature of many advanced ML algorithms—particularly deep neural networks—poses a fundamental challenge to clinical acceptance. Physicians and regulatory agencies demand transparent, explainable decision-making, especially for high-stakes diagnostic applications.

Array-Specific Interpretability Needs: For optical sensing arrays, interpretability is not merely a regulatory requirement but also a scientific necessity. Understanding which spectral features, sensor channels, or colorimetric changes drive diagnostic predictions can reveal underlying biochemical mechanisms and guide sensor optimization. In SERS-based diagnostics, for example, identifying that specific Raman peaks (e.g., nucleic acid signatures at 1600 cm^−1^ or protein amide bands) are responsible for classification provides biological plausibility and strengthens confidence in the diagnostic model. Similarly, for fluorescent arrays, knowing which probe responses are most discriminative can inform probe selection for future sensor designs.

Explainable AI (XAI) Tools: Methods such as SHAP (SHapley Additive exPlanations), LIME (Local Interpretable Model-agnostic Explanations), and integrated gradients can identify which spectral features, sensor channels, or wavelength regions most strongly influence diagnostic predictions [94,110]. Attention mechanisms in deep learning models provide visual saliency maps, highlighting the input regions that the model “attends to” during classification. For SERS-based diagnostics, these techniques have been used to correlate model decisions with known biochemical peaks—such as nucleic acid signatures at 1600 cm^−1^ or protein amide bands—thereby grounding algorithmic outputs in biological plausibility [9].

### 5.4. Sensor Fabrication, Stability, and Scalability

The translation of optical sensing arrays from laboratory prototypes to clinical devices hinges on overcoming fundamental fabrication and stability challenges.

Batch-to-Batch Reproducibility: Even minor variations in sensor fabrication—such as nanoparticle size distribution, surface functionalization density, or substrate morphology—can substantially alter optical responses, particularly at low analyte concentrations [72,97]. Precision manufacturing processes, coupled with batch-specific calibration models, are essential to ensure consistent sensor performance. ML-driven quality control, in which models predict sensor performance from pre-deployment characterization data, offers a promising approach to identify and reject out-of-specification batches.

Material Stability and Shelf Life: Many sensing elements—particularly biological recognition molecules such as antibodies and enzymes—exhibit limited long-term stability, degrading over time or upon exposure to biological fluids [83,111]. This is especially problematic for point-of-care applications where devices may be stored for extended periods before use. Advances in synthetic receptors (aptamers, molecularly imprinted polymers, peptide mimics), protective coatings, and lyophilization techniques are critical to extending sensor shelf life. Encouragingly, ML models can predict degradation kinetics based on storage conditions and initial characterization data, thus permitting proactive calibration adjustments and informing replacement schedules.

Cost-Effectiveness and Scalability: While individual sensor components may be inexpensive, the complete diagnostic system—including instrumentation, consumables, software, and data infrastructure—must demonstrate cost-effectiveness relative to existing diagnostic approaches. Scalable manufacturing processes, quality control protocols, and supply chain reliability are essential for widespread clinical adoption.

### 5.5. Clinical Translation and Point-of-Care Integration

Regulatory Pathways: Optical sensor arrays incorporating ML components face complex regulatory hurdles as software-as-medical-device (SaMD) products [15,112]. Clear validation protocols, clinical trial designs, and post-market surveillance frameworks must be established. Regulatory bodies—including the FDA, European Medicines Agency, and National Medical Products Administration—are formulating specific guidance for AI/ML-enabled medical devices, emphasizing the need for explainability, robustness, and ongoing performance monitoring.

Point-of-Care Integration: Smartphone-based readout, microfluidic integration, and user-friendly device design are critical enablers of decentralized testing [15]. However, these require substantial engineering efforts to balance portability, sensitivity, and robustness [104,113]. Smartphone cameras can capture colorimetric or fluorescent signals, but variations in camera hardware and ambient lighting conditions pose additional challenges for data standardization and ML model performance. Edge computing approaches—in which ML inference is performed locally on low-power devices rather than in the cloud—address latency and privacy concerns while reducing reliance on network connectivity.

Regulatory and Ethical Frameworks: The deployment of ML-based diagnostic sensors must be guided by established ethical and regulatory frameworks, including GDPR, FDA regulations, and ISO 15189 for laboratory quality [13,18]. Federated learning—in which models are trained across multiple institutions without sharing raw patient data—offers a promising solution to privacy concerns while permitting large-scale, diverse training datasets. Explainability techniques such as SHAP not only enhance clinical trust but also align with regulatory requirements for transparency in algorithmic decision-making.

### 5.6. Future Directions

Looking forward, several key research priorities will shape the next generation of ML-assisted optical sensing arrays:

- Multidisciplinary Integration and Convergence: Continued cross-disciplinary collaboration—spanning materials science, analytical chemistry, biology, clinical medicine, and computational science—is essential to address the interconnected challenges of sensor design, data acquisition, algorithmic development, and clinical validation.
- Wearable and Continuous Monitoring Platforms: Advances in flexible electronics, biocompatible materials, and low-power wireless communication are driving the integration of optical sensing arrays into wearable devices. These platforms promise continuous, real-time monitoring of disease biomarkers, moving beyond single time-point diagnostics to dynamic health surveillance.
- Home-Based Self-Testing: Smartphone-readable sensor arrays, coupled with user-friendly mobile applications, could empower patients to monitor chronic conditions at home, reducing healthcare burden, facilitating earlier intervention, and improving quality of life. Challenges include ensuring user compliance, data quality, and connectivity.
- Single-Cell and Spatial Analysis: Extending optical sensing arrays to single-cell or tissue spatial analysis could revolutionize our understanding of disease heterogeneity, revealing distinct cellular subpopulations and enabling more precise, personalized diagnostic strategies.
- Emerging Sensing Modalities: Novel techniques—including photoacoustic imaging, upconversion luminescence, persistent luminescence, and quantum dot-based sensing—offer new dimensions of optical information that could be integrated into array platforms, expanding the range and sensitivity of detectable biomarkers.
- Adaptive and Continuous Learning Systems: Future ML models must be designed to continuously learn from new data as they are deployed, adapting to evolving disease patterns, changing patient populations, and sensor drift. This requires robust online learning algorithms, automated data quality assessment, and human-in-the-loop validation frameworks to ensure safety and reliability.

Collectively, these directions point toward a future where intelligent, portable, and personalized diagnostic systems become a clinical reality, fundamentally transforming how diseases are detected and managed.

## 6. Conclusions

The synergistic integration of ML with optical sensing arrays has substantially advanced the field of disease diagnostics, transforming high-dimensional, multiplexed optical fingerprints into clinically interpretable decisions. This convergence has enabled rapid, sensitive, and cost-effective detection across a wide spectrum of pathologies, from cancers to infectious and neurodegenerative disorders. Colorimetric arrays offer simplicity and smartphone compatibility; fluorescent platforms provide exceptional sensitivity for scarce biomarkers such as exosomes and nucleic acids; and SERS arrays deliver both high sensitivity and rich molecular specificity. Across these modalities, unsupervised learning (e.g., PCA, t-SNE, and UMAP) facilitates pattern discovery and dimensionality reduction; supervised algorithms (SVM, RF, and ANN) enable accurate classification and quantification; and deep learning architectures automatically extract hierarchical features, consistently achieving state-of-the-art performance.

Despite these advances, several obstacles remain. Data scarcity, batch effects, and lack of standardized acquisition protocols compromise model robustness and generalizability. The “black-box” nature of complex algorithms hinders clinical trust and regulatory acceptance, while fabrication reproducibility, material stability, and long-term sensor durability pose practical hurdles for real-world deployment. Furthermore, across the reviewed studies, critical gaps persist in terms of standardized validation protocols, independent external cohort testing, and systematic comparisons to conventional non-ML diagnostic methods—issues that must be addressed to facilitate robust clinical translation. Moreover, the computational expense of training and running deep models, along with ethical considerations regarding patient data privacy and algorithmic fairness, must be carefully addressed.

Looking forward, multidisciplinary collaboration—bridging materials science, analytical chemistry, clinical medicine, and data science—will be essential to overcome these barriers. Standardized data repositories, explainable AI frameworks, and rigorous external validation are critical to building trustworthy diagnostic systems. Advances in flexible electronics, edge computing, and federated learning promise to extend these platforms toward wearable, continuous monitoring and home-based self-testing, ultimately enabling personalized, accessible, and intelligent healthcare. By confronting these challenges head-on, ML-reinforced optical sensing arrays hold immense potential to reshape the diagnostic landscape and improve patient outcomes globally.

## Figures and Tables

**Table 1:** Representative performance metrics of ML-assisted optical sensing arrays.

Sensor Modality	Target Disease/Analyte	ML Algorithm (s)	Accuracy	Sensitivity	Specificity	Key Advantage	Key Limitation	Sample Size	Validation Type	Ref
Colorimetric (Nanozyme-based)	Urinary tract infection	SVM, UMAP	97%	NR	NR	Rapid detection (<1 h), portable, low cost	Limited multiplexing capability	60 clinical samples	Cross-validation	[19]
Colorimetric (AuNP-based)	ESKAPE pathogens (antibiotic resistance)	SVM	89.7%	NR	NR	Fast (20 min), simple readout	Moderate accuracy; requires further optimization	78 clinical isolates	Cross-validation	[69]
Colorimetric (AuNP-based)	Microbial taxonomic identification	ML algorithms	100%	NR	NR	Comparable to gene sequencing	Requires microbial culture for AuNP synthesis	17 microbial species	Cross-validation	[65]
Fluorescent (DNA computing)	Non-small cell lung cancer	SVM-based classifier	90%	NR	NR	Instrument-free molecular computation	Complex DNA probe design; moderate sample size	25 NSCLC patients, 25 healthy	Independent test set	[70]
Fluorescent (Exosome- based)	Breast cancer subtyping	ML classification	High accuracy	NR	NR	Super-resolution colocalization reduces false positives	Requires specialized imaging equipment	4 cell lines; 16 clinical samples	Cross-validation	[71]
Fluorescent (Nanozyme-based)	Tumor exosomes	LDA	100%	NR	NR	High sensitivity; signal amplification	Complex preparation steps	5 cancer cell-derived exosomes	Cross-validation	[66]
SERS (Label-free)	Liver diseases	CNN	97.3–98.6%	NR	NR	Rapid serological detection (<1 min)	Requires precise substrate fabrication	1140 training spectra; 100 external	10-fold CV + external validation set	[67]
SERS (Label-free)	Neuropsychiatric SLE (NPSLE)	ML-assisted	95%	95.9%	93.5%	Non-invasive, high specificity	Requires further clinical validation	73 serum samples (35 SLE, 38 NPSLE)	Cross-validation + blind test	[68]
SERS (Label-free)	Alzheimer’s disease (CSF)	CNN	92%	NR	NR	High reproducibility; early detection potential	Limited sample availability	30 CSF samples	Leave-one-out validation	[72]
SERS (Labeled)	Breast cancer subtyping	ML algorithms	NR	NR	NR	Multiplexed protein analysis	Labeling steps required	3 breast cancer cell lines	Cross-validation	[73]
SERS (Labeled)	Bacterial species identification	PCA-LDA, ML	>98%	NR	NR	No gene amplification or specific biomarkers needed	Surface chemotaxonomy approach still emerging	6 bacterial species	Cross-validation	[74]
Microfluidic Optical	Acute myocardial infarction (cTnI, HDL, LDL)	CatBoost, RF	92% (CatBoost)	NR	NR	Multiplex detection in single test	Requires integration with ML model for full utility	50 AMI cases, 50 controls	Cross-validation	[75]
Microfluidic Optical	Mycobacterium tuberculosis	K-NN	96%	NR	NR	High sensitivity; applicable to clinical diagnostics	Multiple parameters must be analyzed	Simulation-based	Cross-validation	[76]
Wearable Physiological	Stress detection	XGBoost	96.12%	NR	NR	Non-invasive, real-time, robust against noise	External factors affect accuracy; privacy concerns	16 subjects (WESAD dataset)	Cross-validation	[77]

NR: not reported; CV: cross-validation.

**Table 2:** Comprehensive comparison of colorimetric, fluorescent, and SERS sensor arrays for ML-assisted disease diagnosis.

Feature	Colorimetric Arrays	Fluorescent Arrays	SERS Arrays	Ref.
Typical LOD	μM–mM (nanozyme-based: ~40 nM for biothiols)	nM–pM (exosomes: 2.5 × 10^3^ particles/mL)	pM–fM (label-free serum: pM level)	[19,66,72,79,80,96]
Cost per Test	Very low (inexpensive materials, smartphone-compatible)	Moderate (fluorescent probes, optical instrumentation)	High (expensive substrates, Raman spectrometer)	[9,68]
Instrumentation	Smartphone camera, plate reader, UV-Vis spectrophotometer	Fluorometer, fluorescence microscope, microplate reader	Raman spectrometer (confocal/micro), portable Raman systems	[65,67,69]
Sample Preparation	Minimal (direct serum/urine/saliva application)	Often requires labeling (fluorescent dyes, aptamers, antibodies)	May require purification; label-free options available	[65,69,70,74]
Assay Time	Minutes (typically <1 h; some 20–60 min)	Minutes to hours (30–90 min typical)	Minutes (often <20 min; label-free SERS)	[19,22,68,70,96]
Main Applications	POCT, urine analysis, gas sensing, pathogen identification	Exosome detection, nucleic acid assays, protein profiling, microbial sensing	Biomarker fingerprinting, single-cell analysis, cancer subtyping, pathogen identification	[19,22,44,65,66,69,70,74,96]
Data Type	RGB images, absorbance spectra (UV-Vis)	Fluorescence intensity, lifetime, polarization parameters	Raman spectra (high-dimensional, fingerprint-specific)	[19,65,66,67,71,72]
Preferred ML Algorithms	SVM, RF, k-NN, PCA, LDA, UMAP (classical supervised classifiers after simple feature extraction)	ANN, LDA, deep learning (CNN), SVM (time-resolved/ratiometric signals)	CNN, 1D-CNN, SVM, ensemble methods (RF, XGBoost), t-SNE, PCA-LDA	[19,22,32,44,58,59,65,66,67,68,69,71,74,96]

## Data Availability

No new data were created or analyzed in this study. Data sharing is not applicable to this article.

## References

[1] Sathyavathy G., Manimegalai P., Umamakeswari G. (2024). Global Burden of Diseases: Emerging NCDs Burden and Epidemiological Transition.

[2] Blandin Knight S., Crosbie P.A., Balata H., Chudziak J., Hussell T., Dive C. (2017). Progress and Prospects of Early Detection in Lung Cancer. Open Biol..

[3] Zhou B., Chen Z., Zhu H., Chen B., Fang J., Jin X. (2026). Impact of Molecular Rapid Diagnostic Testing on Clinical Outcomes in Bloodstream Infections: A Meta-Analysis. BMC Infect. Dis..

[4] Heidary O., Shojaeifard Z., Akhond M., Fattahi M.J., Ghaderi A., Firuzi O., Hemmateenejad B. (2025). Miniaturized Biomimetic Taste-Smell Fusion Sensor Array for Rapid and Simultaneous Profiling of Volatile and Non-Volatile Blood Plasma Constituents toward Identification of Head and Neck Carcinoma and Breast Cancer. Chem. Eng. J..

[5] Liu Z., Xiang C., Tong Y., Li K.H., Guan X. (2025). Transfer Learning Enhanced Blood Pressure Monitoring Based on Flexible Optical Pulse Sensing Patch. ACS Sens..

[6] Crawford J.M., Shotorbani K., Swanson K. (2025). The Role of the Clinical Laboratory in Diagnostic Stewardship and Population Health. J. Appl. Lab. Med..

[7] Mousavizadegan M., Hosseini M., Mohammadimasoudi M., Guan Y., Xu G. (2024). Machine Learning-Assisted Liquid Crystal Optical Sensor Array Using Cysteine-Functionalized Silver Nanotriangles for Pathogen Detection in Food and Water. ACS Appl. Mater. Interfaces.

[8] Haque M.D., Partho P.K.R., Rafi A.H., Rahman M.M., Mamoon I. (2026). Design and Performance Analysis of Metal-Dielectric Mid-Infrared Refractive Index Sensor with Machine Learning Algorithm for Detecting Anemia and Cancer Cell. Optik.

[9] Fu R., Chen Z., Tian H., Hu J., Bu F., Zheng P., Chi L., Xue L., Jiang Q., Li L. (2025). A Review on the Applications of Machine Learning in Biomaterials, Biomechanics, and Biomanufacturing for Tissue Engineering. Smart Mater. Med..

[10] Zhou S.K., Greenspan H., Davatzikos C., Duncan J.S., Van Ginneken B., Madabhushi A., Prince J.L., Rueckert D., Summers R.M. (2021). A Review of Deep Learning in Medical Imaging: Imaging Traits, Technology Trends, Case Studies with Progress Highlights, and Future Promises. Proc. IEEE.

[11] Jia Z., Holliday E.G., Tang E.C., Russo H.B., Rootes T.R., Luo Y., Yu H., Wang D., Zhang B. (2026). Machine Learning–Driven Nanoparticle–Enhanced Paper Chromogenic Array Sensor Approach for Detecting Sub-Lethally Injured *Salmonella* in Low Moisture Food. Food Res. Int..

[12] Dong M., Li Y., Rai S., Zhang H., Bilotti E., Papageorgiou D.G. (2026). Machine-Learning-Enabled Starch/MXene Moisture Sensing Bioelectronics for Human Monitoring and Interaction. Chem. Eng. J..

[13] Chin W.J., Lim W.Y., Khor S.M., Ramakrishnan N., Chee P.S., Goh C.-H. (2026). Advancement of Machine Learning Algorithms in Biosensors. Clin. Chim. Acta.

[14] Bian Z., Bao T., Sun X., Wang N., Mu Q., Jiang T., Yu Z., Ding J., Wang T., Zhou Q. (2024). Machine Learning Tools to Assist the Synthesis of Antibacterial Carbon Dots. Int. J. Nanomed..

[15] Raza R.S., Fatah S.A., Ameen S.S.M., Omer K.M. (2026). Machine Learning–Empowered Smartphone Platforms for Sustainable Point-of-Care Bacterial Detection. Talanta.

[16] Hu J., Li M., Yang R., Bai C., Chen B., Shen J., Zhang W., Tang W., Li Z. (2026). Machine Learning-Assisted Arginine Modified Copper Nanozyme Sensing Array for Detection of Antioxidant Phenolics. Microchem. J..

[17] Gao Z., Song Y., Hsiao T.Y., He J., Wang C., Shen J., MacLachlan A., Dai S., Singer B.H., Kurabayashi K. (2021). Machine-Learning-Assisted Microfluidic Nanoplasmonic Digital Immunoassay for Cytokine Storm Profiling in COVID-19 Patients. ACS Nano.

[18] Baluta S., Suresh V., Chmielowska M., Smeesters L., Cabaj J. (2026). Improving Clinical Diagnostics and Patient Care through Artificial Intelligence and Biosensor Technologies. ACS Omega.

[19] Yang J., Li G., Chen S., Su X., Xu D., Zhai Y., Liu Y., Hu G., Guo C., Yang H.B. (2024). Machine Learning-Assistant Colorimetric Sensor Arrays for Intelligent and Rapid Diagnosis of Urinary Tract Infection. ACS Sens..

[20] Xia H., Xiong L., Huang R., Liu N., Muhammad M., Hong J., Huang Q. (2026). Machine Learning-Assisted SERS-Based Dual-Aptamer Biosensor for Ultrasensitive Clinical Screening of Breast Cancer. Spectrochim. Acta Part A Mol. Biomol. Spectrosc..

[21] Ma Q., Zhang M., Zhang C., Teng X., Yang L., Tian Y., Wang J., Han D., Tan W. (2022). An Automated DNA Computing Platform for Rapid Etiological Diagnostics. Sci. Adv..

[22] Liu S., Hu N., Hu J., Li W., Wu S., Ma X., Huo Y.-X., Lu Y. (2025). DNA-Mediated Bioinspired MXene Gas Sensor Array with Machine Learning for Noninvasive Cancer Recognition. ACS Nano.

[23] Bansal K., Kaur B., Boddu S., Kumar S., Marques C. (2025). Machine Learning Integration in Optical Sensing: Trends, Challenges, and Biomedical Applications. IEEE Sens. Rev..

[24] Cialla-May D., Bonifacio A., Markin A., Markina N., Fornasaro S., Dwivedi A., Dib T., Farnesi E., Liu C., Ghosh A. (2024). Recent Advances of Surface Enhanced Raman Spectroscopy (SERS) in Optical Biosensing. TrAC Trends Anal. Chem..

[25] Li Z., Suslick K.S. (2021). The Optoelectronic Nose. Acc. Chem. Res..

[26] Peveler W.J. (2024). Food for Thought: Optical Sensor Arrays and Machine Learning for the Food and Beverage Industry. ACS Sens..

[27] Tian C., Shin S., Cho Y., Song Y., Cho S.-Y. (2024). High Spatiotemporal Precision Mapping of Optical Nanosensor Array Using Machine Learning. ACS Sens..

[28] Wen T., Che R., Chen H., Jin C., Wang H., Huang K.-J., Diao K., Tan X. (2026). An Intelligent Nanozyme-Enhanced Triple-Modal Detection Platform Integrated with Machine Learning for Self-Validating Pathogen Monitoring. Sens. Actuators B Chem..

[29] Xue X., Zheng F., Luo Y., Chen W., Gao Y., Wei K. (2025). A Multifunctional Pt/DMSN Nanozyme-Based Colorimetric-Fluorescence Sensing Platform for Breast Cancer Detection. Microchim. Acta.

[30] Zhang S., Zhu W., Zhang J., Zhang X., Wang F. (2025). Perovskite Quantum Dot-Based Fluorescent Sensor Array Coupled with Machine Learning for Rapid Pathogenic Bacteria Detection and Identification. Chem. Eng. J..

[31] Wu S., Yu Z., Zhao J., Wang Y., Nan J., Xu L., Li X., Yang L., Dong S. (2025). Fluorescent Nanozyme Based on Bimetallic Fe/Co-Doped Carbon Dots for Dual-Mode Detection of Ascorbic Acid and Hydrogen Peroxide. Anal. Chem..

[32] Kim H., Lee S., Lee K.W., Kim E.S., Kim H.-M., Im H., Yoon H.C., Ko J., Kim E. (2023). Indolizine-Based Fluorescent Compounds Array for Noninvasive Monitoring of Glucose in Bio-Fluids Using on-Device Machine Learning. Dye. Pigment..

[33] Sahli C., Kenry (2024). Enhancing Nanomaterial-Based Optical Spectroscopic Detection of Cancer through Machine Learning. ACS Mater. Lett..

[34] Resmi A.N., Nazeer S.S., Dhushyandhun M.E., Paul W., Chacko B.P., Menon R.N., Jayasree R.S. (2024). Ultrasensitive Detection of Blood-Based Alzheimer’s Disease Biomarkers: A Comprehensive SERS-Immunoassay Platform Enhanced by Machine Learning. ACS Chem. Neurosci..

[35] Qin Y.-F., Lu X.-Y., Shi Z., Huang Q.-S., Wang X., Ren B., Cui L. (2022). Deep Learning-Enabled Raman Spectroscopic Identification of Pathogen-Derived Extracellular Vesicles and the Biogenesis Process. Anal. Chem..

[36] Bai Z. (2024). Artificial Intelligence-Assisted Identification and Validation of Anti-Aging Biomarkers. Acad. J. Med. Health Sci..

[37] Wasilewski T., Kamysz W., Gębicki J. (2024). AI-Assisted Detection of Biomarkers by Sensors and Biosensors for Early Diagnosis and Monitoring. Biosensors.

[38] Wang Y., Feng Y., Zhang B., Upadhyay A., Xiao Z., Luo Y. (2024). Machine Learning-Supported Sensor Array for Multiplexed Foodborne Pathogenic Bacteria Detection and Identification. Trends Food Sci. Technol..

[39] Pan H., Chen X., Han R., He X., Xu J., Zhang Y., He S., Xu M., Wang M., Xu X. (2026). Machine Learning-Assisted Regulator-Mediated Single Nanozyme Sensor Array Rapid Detection of Proteins and Nutritional Supplements. Sens. Actuators B Chem..

[40] Lu S., Yang J., Gu Y., He D., Wu H., Sun W., Xu D., Li C., Guo C. (2024). Advances in Machine Learning Processing of Big Data from Disease Diagnosis Sensors. ACS Sens..

[41] Kangas M.J., Burks R.M., Atwater J., Lukowicz R.M., Garver B., Holmes A.E. (2018). Comparative Chemometric Analysis for Classification of Acids and Bases via a Colorimetric Sensor Array. J. Chemom..

[42] Liu M., Wang T., Zhang Q., Pan C., Liu S., Chen Y., Lin D., Feng S. (2024). An Outlier Removal Method Based on PCA-DBSCAN for Blood-SERS Data Analysis. Anal. Methods.

[43] Yaltaye M.W., Zhao Y., Zhan K., Bozo E., Xin P.-L., Farrahi V., De Angelis F., Huang J.-A. (2025). Single-Molecule SERS Detection of Phosphorylation in Serine and Tyrosine Using Deep Learning-Assisted Plasmonic Nanopore. J. Phys. Chem. Lett..

[44] Rodriguez-Nieves A.L., Taylor M.L., Wilson R., Eldridge B.K., Nawalage S., Annamer A., Miller H.G., Alle M.R., Gomrok S., Zhang D. (2024). Multiplexed Surface Protein Detection and Cancer Classification Using Gap-Enhanced Magnetic–Plasmonic Core–Shell Raman Nanotags and Machine Learning Algorithm. ACS Appl. Mater. Interfaces.

[45] García-Astrain C., Henriksen-Lacey M., Lenzi E., Renero-Lecuna C., Langer J., Piñeiro P., Molina-Martínez B., Plou J., Jimenez De Aberasturi D., Liz-Marzán L.M. (2024). A Scaffold-Assisted 3D Cancer Cell Model for Surface-Enhanced Raman Scattering-Based Real-Time Sensing and Imaging. ACS Nano.

[46] Liu F., Liu J., Luo Y., Wu S., Liu X., Chen H., Luo Z., Yuan H., Shen F., Zhu F. (2024). A Single-Cell Metabolic Profiling Characterizes Human Aging via SlipChip-SERS. Adv. Sci..

[47] Aina O.E., Zine N., Raffin G., Jaffrezic-Renault N., Elaissari A., Errachid A. (2024). Integrated Breath Analysis Technologies: Current Advances and Future Prospects. TrAC Trends Anal. Chem..

[48] Lu S., Yang J., Xing H., Chang Y., Sun J., Guo C., Yang X. (2023). FRET Cascade miRNA Addition Probe from Non-Crosstalk DNA Photonic Wire Assisted with Clustering Algorithm for Early Diagnosis of Acute Myocardial Infarction. Biosens. Bioelectron..

[49] Joshi P., Mondal P.P. (2021). Single-Molecule Clustering for Super-Resolution Optical Fluorescence Microscopy. Photonics.

[50] Lalman C., Yang Y., Walker J.L. (2025). Artificial Intelligence in Ocular Transcriptomics: Applications of Unsupervised and Supervised Learning. Cells.

[51] Wang Y., Ding L., Du M., Zhang J., Ping Q., Wang L., Wang A., Zhao H., Yu X. (2026). Integrated Colorimetric Immunoassay for PSA Using Confined Pt Nanozymes and Smartphone-Assisted Machine Learning Analysis. Sens. Actuators B Chem..

[52] Zhen L., Li J., Bi Z., Zhang X., Jin P., Huang H., Li Y. (2025). A Three-Channel Nanozyme Sensor Array Combined with Machine Learning Algorithm for the Differentiation and Detection of Aminoglycoside Antibiotics. Chem. Eng. J..

[53] Han Q., Chen X., Huang H., Li Y. (2026). Machine Learning-Assisted Colorimetric Sensor Array Based on Laccase-like Nanozymes for Identification of Eleven Halogenated Phenolic Pollutants over a Wide Concentration Range. Microchem. J..

[54] Yan J., Liu R., Yang W., Qu C., Jiang M. (2026). Machine Learning-Assisted Plasmonic Sensing Using Au@Ag–Ti_3_C_2_T_x_ MXene/Au Hybrid Interfaces for High-Efficiency and Ultrasensitive microRNA Detection. Biosens. Bioelectron..

[55] Winchester L.M., Harshfield E.L., Shi L., Badhwar A., Khleifat A.A., Clarke N., Dehsarvi A., Lengyel I., Lourida I., Madan C.R. (2023). Artificial Intelligence for Biomarker Discovery in Alzheimer’s Disease and Dementia. Alzheimer’s Dement..

[56] Wang J., Zhou Z., Luo Y., Xu T., Xu L., Zhang X. (2024). Machine Learning-Assisted Janus Colorimetric Face Mask for Breath Ammonia Analysis. Anal. Chem..

[57] Kumar B.N., Kallam S., Sharan P., Kumaresan M. (2025). Integrating Deep Learning with Photonic SPR Biosensors for Pathogen and Biomarker Detection. Proceedings of the 2025 12th International Conference on Computing for Sustainable Global Development (INDIACom), Delhi, India, 2 April 2025.

[58] Cheng N., Chen D., Lou B., Fu J., Wang H. (2021). A Biosensing Method for the Direct Serological Detection of Liver Diseases by Integrating a SERS-Based Sensor and a CNN Classifier. Biosens. Bioelectron..

[59] Xiong C.-C., Zhu S.-S., Yan D.-H., Yao Y.-D., Zhang Z., Zhang G.-J., Chen S. (2023). Rapid and Precise Detection of Cancers via Label-Free SERS and Deep Learning. Anal. Bioanal. Chem..

[60] Hayat M.T., Allawi Y.M., Alamro W., Sultan S.M., Abadleh A., Kang H., Zreikat A.I. (2025). A Hybrid Convolutional Neural Network–Long Short-Term Memory (CNN–LSTM)–Attention Model Architecture for Precise Medical Image Analysis and Disease Diagnosis. Diagnostics.

[61] Hanley J.A., McNeil B.J. (1982). The Meaning and Use of the Area under a Receiver Operating Characteristic (ROC) Curve. Radiology.

[62] Davis J., Goadrich M. (2006). The Relationship Between Precision-Recall and ROC Curves. Proceedings of the 23rd International Conference on Machine Learning—ICML ’06.

[63] Sokolova M., Lapalme G. (2009). A Systematic Analysis of Performance Measures for Classification Tasks. Inf. Process. Manag..

[64] Chicco D., Jurman G. (2020). The Advantages of the Matthews Correlation Coefficient (MCC) over F1 Score and Accuracy in Binary Classification Evaluation. BMC Genom..

[65] Yu K., Lu S., Qiu K., Zhang Y., Sun A., Gong S., Wang K., Gao X., Xu X., Wang H. (2025). Biomimetic Analysis of Neurotransmitters for Disease Diagnosis through Light-Driven Nanozyme Sensor Array and Machine Learning. Adv. Sci..

[66] Liu M.-X., Zhang H., Zhang X.-W., Chen S., Yu Y.-L., Wang J.-H. (2021). Nanozyme Sensor Array Plus Solvent-Mediated Signal Amplification Strategy for Ultrasensitive Ratiometric Fluorescence Detection of Exosomal Proteins and Cancer Identification. Anal. Chem..

[67] Cheng N., Fu J., Chen D., Chen S., Wang H. (2021). An Antibody-Free Liver Cancer Screening Approach Based on Nanoplasmonics Biosensing Chips via Spectrum-Based Deep Learning. NanoImpact.

[68] Yu T., Fu Y., He J., Zhang J., Xianyu Y. (2023). Identification of Antibiotic Resistance in ESKAPE Pathogens through Plasmonic Nanosensors and Machine Learning. ACS Nano.

[69] Yang L., Tang Q., Zhang M., Tian Y., Chen X., Xu R., Ma Q., Guo P., Zhang C., Han D. (2024). A Spatially Localized DNA Linear Classifier for Cancer Diagnosis. Nat. Commun..

[70] Wei J., Zhu K., Wang T., Qi T., Wang Z., Li J., Zong S., Cui Y. (2024). Highly Accurate Profiling of Exosome Phenotypes Using Super-Resolution Tricolor Fluorescence Co-Localization. ACS Nano.

[71] Mi Y., Li X., Zeng X., Cai Y., Sun X., Yan Y., Jiang Y. (2024). Diagnosis of Neuropsychiatric Systemic Lupus Erythematosus by Label-Free Serum Microsphere-Coupled SERS Fingerprints with Machine Learning. Biosens. Bioelectron..

[72] Yu X., Srivastava S., Huang S., Hayden E., Teplow D., Xie Y.-H. (2022). The Feasibility of Early Alzheimer’s Disease Diagnosis Using a Neural Network Hybrid Platform. Biosensors.

[73] Wang J., Cong L., Shi W., Xu W., Xu S. (2023). Single-Cell Analysis and Classification According to Multiplexed Proteins via Microdroplet-Based Self-Driven Magnetic Surface-Enhanced Raman Spectroscopy Platforms Assisted with Machine Learning Algorithms. Anal. Chem..

[74] Leong S.X., Tan E.X., Han X., Luhung I., Aung N.W., Nguyen L.B.T., Tan S.Y., Li H., Phang I.Y., Schuster S. (2023). Surface-Enhanced Raman Scattering-Based Surface Chemotaxonomy: Combining Bacteria Extracellular Matrices and Machine Learning for Rapid and Universal Species Identification. ACS Nano.

[75] Low J.S.Y., Thevarajah T.M., Chang S.W., Khor S.M. (2023). Paper-Based Multiplexed Colorimetric Biosensing of Cardiac and Lipid Biomarkers Integrated with Machine Learning for Accurate Acute Myocardial Infarction Early Diagnosis and Prognosis. Sens. Actuators B Chem..

[76] Parmar J., Patel S.K., Katkar V., Natesan A. (2023). Graphene-Based Refractive Index Sensor Using Machine Learning for Detection of Mycobacterium Tuberculosis Bacteria. IEEE Trans. NanoBiosci..

[77] Nazeer M., Salagrama S., Kumar P., Sharma K., Parashar D., Qayyum M., Patil G. (2024). Improved Method for Stress Detection Using Bio-Sensor Technology and Machine Learning Algorithms. MethodsX.

[78] Yadav S., Sarkar A., Shivalkar S., Fatima F., Thakur S.K., Chaudhary A., Samanta S.K., Sahoo A.K. (2026). Recent Advances in Metal-Organic Framework-Based Nanozymes for Cancer Theranostics Driven by Synthetic Innovation and Machine Learning Design. Colloids Surf. B Biointerfaces.

[79] Wang M., Zhang H., Yan S., Zhou Y., Guo X., An D., Zhong W., Zhang Y. (2025). Fabrication of MOF-Based Nanozyme Sensor Arrays and Their Application in Disease Diagnosis. Coord. Chem. Rev..

[80] Wei M., Yang M., Leng H., Shu Y. (2025). A Novel Intelligent Sensing Strategy: Integration of Metal-Doped Carbon Dots Nanozymes and Machine Learning for Rapid Screening of Biothiols in Disease. Sens. Actuators B Chem..

[81] Shao Z., Zhang J., Jin Z., Qu F., Du C., Ming Y., Li L., Xue Y., Chen Q., He L. (2026). Machine Learning-Enabled Microfluidic Ratiometric Fluorescence Sensor Array Based on Lanthanide-Gold Nanoclusters for Visual Detection of Multicomponent Antibiotics. Biosens. Bioelectron..

[82] Yu T., Su S., Hu J., Zhang J., Xianyu Y. (2022). A New Strategy for Microbial Taxonomic Identification through Micro-Biosynthetic Gold Nanoparticles and Machine Learning. Adv. Mater..

[83] Wang X., Yang T., Wang J.-H. (2024). Recent Advances in Sensor Arrays Aided by Machine Learning for Pathogen Identification. Sens. Diagn..

[84] Wang X., Li H., Yang J., Wu C., Chen M., Wang J., Yang T. (2024). Chemical Nose Strategy with Metabolic Labeling and “Antibiotic-Responsive Spectrum” Enables Accurate and Rapid Pathogen Identification. Anal. Chem..

[85] Song H., Li M., Li R., Cheng J., Lin H., Qi R., Yuan H., Bai H. (2025). Statistical Learning-Assisted Dual-Signal Sensing Arrays Based on Conjugated Molecules for Pathogen Detection and Identification. ACS Appl. Mater. Interfaces.

[86] Wang Y., Feng Y., Xiao Z., Luo Y. (2025). Machine Learning Supported Single-Stranded DNA Sensor Array for Multiple Foodborne Pathogenic and Spoilage Bacteria Identification in Milk. Food Chem..

[87] Vafadar A., Maleki M., Ehtiati S., Moghadam H.Z., Savardashtaki A. (2026). Optical Biosensors for Detection of Exosomes. Clin. Chim. Acta.

[88] Lin X., Zhu J., Shen J., Zhang Y., Zhu J. (2024). Advances in Exosome Plasmonic Sensing: Device Integration Strategies and AI-Aided Diagnosis. Biosens. Bioelectron..

[89] Qu X., Lu B., Gao C., Zhao W., Zeng Y., Wu S., Ji C., Li G. (2026). AI-Enabled New Sensing Technology: Colorimetric Analysis of Exosomes for Precise Diagnosis of Breast Cancer. Chem. Sci..

[90] Huang S., Guo W., Sun H., Cui B., Fang Y. (2026). Metabolic Fingerprint-Mediated Chemical Nose Strategy: Precise Identification of Foodborne Bacteria Based on a 4-MU Derivative Fluorescence Array. Anal. Chem..

[91] Yu Y., Ni W., Hu Q., Li H., Zhang Y., Gao X., Zhou L., Zhang S., Ma S., Zhang Y. (2024). A Dual Fluorescence Turn-On Sensor Array Formed by Poly(*Para* -aryleneethynylene) and Aggregation-Induced Emission Fluorophores for Sensitive Multiplexed Bacterial Recognition. Angew. Chem. Int. Ed..

[92] Hu C., Guan M., Mi F., Zhang S., Geng P. (2026). Multimodal SERS Biosensing Platforms: Emerging Opportunities for Ultrasensitive Biomarker Detection and Intelligent Diagnostics. ACS Sens..

[93] Li X., Wang N., Sun R., Li Y., Xu J., Tong J., Qian H., Li R., Lin S., Li J. (2026). Advances in SERS Technology for Molecular Immunophenotyping. TrAC Trends Anal. Chem..

[94] Chen B., Sun H., Gao J., Chen Z., Qiu X. (2025). Machine Learning-Enhanced SERS Diagnostics: Accelerating the AI-Powered Transition from Laboratory Discoveries to Clinical Practice. Comput. Biol. Med..

[95] Dong X., Zhao Z., Zheng Y., Song L., Chen T., Wang W., Zhang Z. (2026). SERS–AI for Biomedical Applications: From Single Spectrum to Multidimensional Analysis. TrAC Trends Anal. Chem..

[96] Diao X., Li X., Hou S., Li H., Qi G., Jin Y. (2023). Machine Learning-Based Label-Free SERS Profiling of Exosomes for Accurate Fuzzy Diagnosis of Cancer and Dynamic Monitoring of Drug Therapeutic Processes. Anal. Chem..

[97] Mpofu K., Chauke S., Thwala L., Mthunzi-Kufa P. (2025). Aptamers and Antibodies in Optical Biosensing. Discov. Chem..

[98] Ge Y., Wang H., Ji T. (2026). Molecular Imprinting-Based Pseudo-ELISA: Recent Advances. Analyst.

[99] Mohan A., Roy I. (2025). Exploring the Diagnostic Landscape: Portable Aptasensors in Point-of-Care Testing. Anal. Biochem..

[100] Cetinkaya A., Isa A., Piskin E., Ozkan S.A. (2026). Integrating Aptamers- and Molecularly Imprinted Polymers in Biosensing Platforms for Cancer Biomarker Detection: Trends and Perspectives. TrAC Trends Anal. Chem..

[101] Jiang Y., Lv S., Zhou Z., Zhang Y., Wang L., Liu J., Sun Y., Zhu M., Wu S. (2026). Machine Learning-Driven MOF-on-MOF Luminescent Array for Multiplexed Biomarker Recognition in Depression Diagnostics and Information Anticounterfeiting. Chem. Eng. J..

[102] Liu J., Chen X., Diao Q., Tang Z., Niu X. (2025). Machine-Learning-Assisted Nanozyme-Based Sensor Arrays: Construction, Empowerment, and Applications. Biosensors.

[103] Gu H., Jiang K., Yu F., Wang L., Yang X., Li X., Jiang Y., Lü W., Sun X. (2024). Multifunctional Human–Computer Interaction System Based on Deep Learning-Assisted Strain Sensing Array. ACS Appl. Mater. Interfaces.

[104] Du J.-Q., Luo W.-C., Zhang J.-T., Li Q.-Y., Bao L.-N., Jiang M., Yu X., Xu L. (2024). Machine Learning-Assisted Fluorescence/Fluorescence Colorimetric Sensor Array for Discriminating Amyloid Fibrils. Sens. Actuators B Chem..

[105] Kashtiaray A., Karimi M., Ghafori-Gorab M., Maleki A. (2025). A Comprehensive Review on the Recent Applications of Nanozymes in Breast Cancer Therapy and Diagnosis. Mater. Adv..

[106] Shanmugasundaram A., Abdullah A., Lee H., Munirathinam K., Paeng C., Anand R., Woo J., Lee H., Yun G.J., Yim C. (2026). Nanostructured Composite *E*-Nose for Selective Exhaled Nitric Oxide Detection and Machine Learning-Assisted Respiratory Disease Diagnosis. Chem. Eng. J..

[107] Shi X., Wu Y., Hu G., Li Y. (2025). Machine Learning-Assisted Lanthanide Fluorescence Sensor Array Based on pH Regulation for Pattern Recognition of Metal Ions in Biological Fluids. J. Phys. Chem. C.

[108] Karim M.M., Lasker T. (2025). Electrochemical Biosensors for Cancer Biomarker Detection: Basic Concept, Design Strategy and Cutting-Edge Development. Electrochem. Sci. Adv..

[109] Pandit S., Banerjee T., Srivastava I., Nie S., Pan D. (2019). Machine Learning-Assisted Array-Based Biomolecular Sensing Using Surface-Functionalized Carbon Dots. ACS Sens..

[110] Hossain M.S., Shorfuzzaman M. (2025). Early Diabetic Retinopathy Cyber-Physical Detection System Using Attention-Guided Deep CNN Fusion. IEEE Trans. Netw. Sci. Eng..

[111] Jin X., Qu M., Huo F., Yin C. (2026). Metal-Doped Carbon Dots Nanozymes: From Structural Regulation to Enzyme-Mimetic Mechanisms and Applications. Dye. Pigment..

[112] Zhou J., Wu L., Wyttenbach M., Lennon R., Liu Y., Christian M., Scherr S., Charara M., Kommalapati R., Blum J. (2026). Microneedle Array Platforms for Drug Delivery and Biomarker Sensing: From Skin Mechanics Guided Design to Scalable Manufacture for Clinical Utility. J. Control. Release.

[113] Schöttle M., Tran T., Oberhofer H., Retsch M. (2023). Machine Learning Enabled Image Analysis of Time-Temperature Sensing Colloidal Arrays. Adv. Sci..

